# Targeted radioligand therapy: physics and biology, internal dosimetry and other practical aspects during ^177^Lu/^225^Ac treatment in neuroendocrine tumors and metastatic prostate cancer

**DOI:** 10.7150/thno.107963

**Published:** 2025-03-18

**Authors:** Habibollah Dadgar, Ali Pashazadeh, Nasim Norouzbeigi, Majid Assadi, Batool Al-balooshi, Richard P. Baum, Akram Al-Ibraheem, Mohamad Haidar, Mohsen Beheshti, Parham Geramifar, Reza Vali, Seyed Mohammadi, Swagat Dash, Vindhya Malasani, Andrea Cimini, Maria Ricci, Abdulredha A. Esmail, Sarah Murad, Fahad Marafi, Giorgio Treglia, Aysar Najeh Khalaf, Farah M. Anwar, Sharjeel Usmani, Yehia Omar, Haider Muhsin, Igore E. Tyurin, Andrew Zakhary, Sahar Al-Sebaie, Danny Mena Cortes, Maryam AlHashim, Hossein Arabi, Habib Zaidi

**Affiliations:** 1 Cancer Research Center, RAZAVI Hospital, Imam Reza International University, Mashhad, Iran.; 2 Department of Nuclear Medicine, Mainz University Hospital, Mainz, Germany.; 3 The Persian Gulf Nuclear Medicine Research Center, Department of Molecular Imaging and Radionuclide Therapy (MIRT), Bushehr Medical University Hospital, Bushehr University of Medical Sciences, Bushehr, Iran.; 4 Dubai Nuclear medicine & Molecular imaging Center- Dubai Academic Health corporation- DAHC, United Arab Emirates.; 5 CURANOSTICUM Wiesbaden-Frankfurt, Center for Advanced Radiomolecular Precision Oncology, Wiesbaden, Germany.; 6 Department of Nuclear Medicine, King Hussein Cancer Center, Amman, Jordan.; 7 Department of Clinical Radiology, American University of Beirut, Beirut, Lebanon.; 8 Division of Molecular Imaging and Theranostics, Department of Nuclear Medicine & Endocrinology, University Hospital, Paracelsus Medical University Salzburg, A-5020 Salzburg, Austria.; 9 Research Center for Nuclear Medicine, Tehran University of Medical Sciences, Tehran, Iran.; 10 Nuclear Medicine department, University of Toronto, Hospital for Sick Children, Toronto, Ontario, Canada.; 11 Hospital & Health Care Professional, Pittsburgh Medical Center, Nuclear Medicine department, Pittsburg, USA.; 12 Department of Nuclear Medicine and Molecular Theranostics, Sarvodaya Hospital, Sector 8, Faridabad, Haryana, India.; 13 Nuclear Medicine Unit, St. Salvatore Hospital, 67100 L'Aquila, Italy.; 14 Nuclear Medicine Unit, Cardarelli Hospital, 86100 Campobasso, Italy.; 15 Nuclear Medicine Department, Kuwait Cancer Control Center, Kuwait City, Kuwait.; 16 Jaber Alahmad Center of Nuclear Medicine and Molecular Imaging, Kuwait City, Kuwait.; 17 Division of Nuclear Medicine, Imaging Institute of Southern Switzerland, Ente Ospedaliero Cantonale, Bellinzona, Switzerland.; 18 Biomedical Sciences, Universitá della Svizzera italiana, Lugano, Switzerland.; 19 Nuclear Medicine Department, Warith International Cancer Institute, Karbala, Iraq.; 20 Department of Nuclear Medicine Sultan Qaboos Comprehensive Cancer Care and Research Center (SQCCCRC), Seeb, Oman.; 21 PET-CT department at Misr Radiology Center, Heliopolis, Egypt.; 22 Nuclear Medicine department, Amir Al-Momineen Specialty Hospital, Al-Najaf Governorate, Iraq.; 23 MOH Russia, Russian Medical Academy of Continuous Professional Education of the Ministry of Healthcare of the Russian Federation, Russia.; 24 Ministry of National Gaurds Health Services, Jeddah, Saudi Arabia.; 25 Oncologia San Jose, UroTeragLATAM, Mexico.; 26 Radiology Department, College of Medicine, Imam Abdulrahman Bin Faisal University, King Faisal Ibn Abd Aziz Rd, Dammam 34212, Saudi Arabia.; 27 Medical Imaging Services Center, King Fahad Specialist Hospital Dammam, Dammam 32253, Saudi Arabia.; 28 Division of Nuclear Medicine and Molecular Imaging, Diagnostic Department, Geneva University Hospital, Geneva, Switzerland.; 29 Department of Nuclear Medicine and Molecular Imaging, University of Groningen, University Medical Center Groningen, Groningen, Netherlands.; 30 Department of Nuclear Medicine, University of Southern Denmark, Odense, Denmark.; 31 University Research and Innovation Center, Óbuda University, Budapest, Hungary.

**Keywords:** Radiopharmaceutical therapies, theranostics, DNA damage, dosimetry, ^177^Lu, ^225^Ac.

## Abstract

Radioligand therapy (RLT) has garnered significant attention due to the recent emergence of innovative and effective theranostic agents, which showed promising therapeutic and prognostic results in various cancers. Moreover, understanding the interaction between different types of radiation and biological tissues is essential for optimizing therapeutic interventions These concepts directly apply to clinical RLTs and play a crucial role in determining the efficacy and toxicity profile of different radiopharmaceutical agents. Personalized dosimetry is a powerful tool that aids in estimating patient-specific absorbed doses in both tumors and normal organs. Dosimetry in RLT is an area of active investigation, as our current understanding of the relationship between absorbed dose and tissue damage is primarily derived from external-beam radiation therapy. Further research is necessary to comprehensively comprehend this relationship in the context of RLTs. In the present review, we present a thorough examination of the involvement of ^177^Lu/^225^Ac radioisotopes in the induction of direct and indirect DNA damage, as well as their influence on the initiation of DNA repair mechanisms in cancer cells of neuroendocrine tumors and metastatic prostate cancer. Current data indicate that high-energy α-emitter radioisotopes can directly impact DNA structure by causing ionization, leading to the formation of ionized atoms or molecules. This ionization process predominantly leads to the formation of irreparable and intricate double-strand breaks (DSBs). On the other hand, the majority of DNA damage caused by β-emitter radioisotopes is indirect, as it involves the production of free radicals and subsequent chemical reactions. Beta particles themselves can also physically interact with the DNA molecule, resulting in single-strand breaks (SSBs) and potentially reversible DSBs.

## Introduction

Nuclear medicine has experienced significant progress in recent years regarding the identification and application of various radiopharmaceuticals for diagnostic and therapeutic purposes. These advancements have been primarily driven by the development of hybrid imaging modalities like SPECT/CT, PET/CT, and PET/MR, which have significantly enhanced the field's capabilities. The evolution of nuclear medicine towards personalized approaches from traditional treatments has been a notable outcome of these technological advancements. The term "Theranostics" derived from the combination of "therapeutic" and "diagnostic," has emerged to describe this integrated approach in current medical practice. Theranostics involves the imaging of tumor cells through the use of a gamma-emitting radiopharmaceutical that targets specific receptors, combined with a therapeutic radionuclide, such as lutetium-177 (^177^Lu), yttrium-90 (^90^Y), or Actinium-225 (^225^Ac), to effectively eliminate the tumor cells while saving healthy tissues and organs.

This innovative approach represents a significant shift in the treatment paradigm towards more precise and targeted therapies in nuclear medicine 1, 2. Currently, nuclear theranostics is used in the treatment of neuroendocrine tumors (NETs) with the use of ^68^Ga/^177^Lu-DOTA-peptides as well as in prostate carcinoma with ^68^Ga/^18^F-PSMA and ^177^Lu/^225^Ac-PSMA ligands. Individual dose calculation, referred to as dosimetry, is crucial for ensuring the effectiveness and safety of RLT. It is crucial to strike a balance to prevent both under- and over-treatment, while also minimizing toxicity 3-6. The field of theranostics in the modern era has successfully merged various specialized disciplines such as nuclear medicine, molecular biology, immunology, genomics, radiomics, artificial intelligence (AI), and more 7-9. This integration has played a crucial role in the advancement of personalized and precision medicine, allowing for a more cohesive and comprehensive approach to healthcare 10.

This article presents an overview of the core principles of theranostics within the realm of Radioligand therapy, highlighting the interconnection between precision medicine, AI, biology and internal dosimetry in theranostics. A solid grasp of fundamental physics and radiation biology is imperative as a knowledge base to comprehend the variables that impact therapeutic outcomes and the potential toxicities associated with therapy, emphasizing the importance of personalized dosimetry in customizing RLTs for individual patients. Furthermore, a thorough exploration of the utilization of AI in imaging and internal dosimetry within both tumor sites and organs at risk (OARs) is essential, given its pivotal role in forecasting tumor response and overall prognosis in the context of RLT.

This review is designed as a comprehensive, foundational resource in radio-theranostics. Recognizing the multidisciplinary nature of the field, our aim is to provide an integrated overview that is accessible to nuclear medicine technologists, medical physicists, and physicians. For readers seeking a more detailed exploration of specific subtopics, we have included targeted references and subheadings throughout the manuscript that direct to more specialized literature.

## Personalized nuclear medicine

Personalized medicine, driven by molecular imaging and theranostics, has emerged as a fundamental approach in tailoring treatment plans to suit the unique needs of each patient. The integration of these advanced technologies has revolutionized the field of medicine, thereby enabling highly precise and effective therapeutic interventions based on individual characteristics and responses 11. Over the past few decades, there has been a notable transition towards personalized medicine, with the goal of reducing unnecessary and expensive treatments, while also improving patient care by focusing on better target localization and treatment strategies 11-13. Diagnostic imaging plays a crucial role in visualizing and localization therapeutic targets, allowing for the identification of specific treatment areas. By utilizing diagnostic scans, healthcare professionals can predict and monitor treatment outcomes, ensuring that the intended sites receive proper treatment during therapy. Additionally, diagnostic imaging aids in determining the most effective treatment strategy.

**Figure 1:**
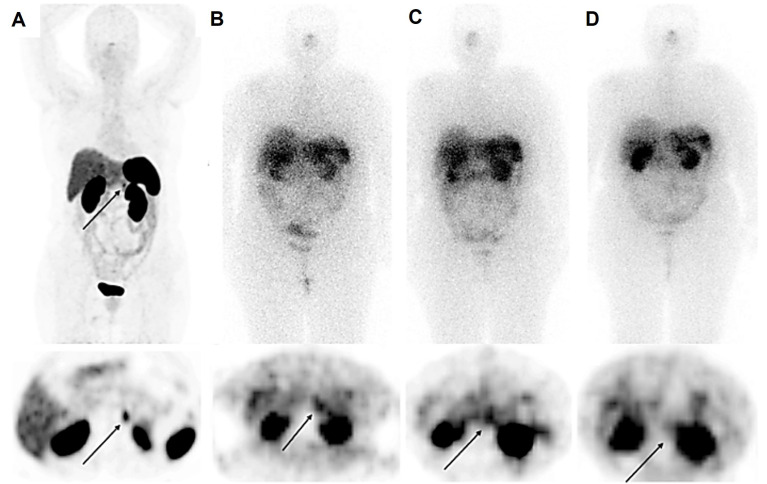
A 56-year-old female with raised calcitonin levels following thyroidectomy, and therefore suspected of recurrent medullary thyroid carcinoma (MTC), underwent ^68^Ga-DOTATATE PET/CT, which showed radiotracer uptake in the pancreatic tail (SUV_max_ = 10.5) (A). The patient underwent 3 cycles of PRRT with ^177^Lu-DOTATATE (10.5 GBq). Post-treatment scintigraphy after the first (B) and second (C) cycles showed an accumulation of radiotracer in the lesions, which was significantly reduced in the third cycle (D), revealing partial response.

**Figure 2:**
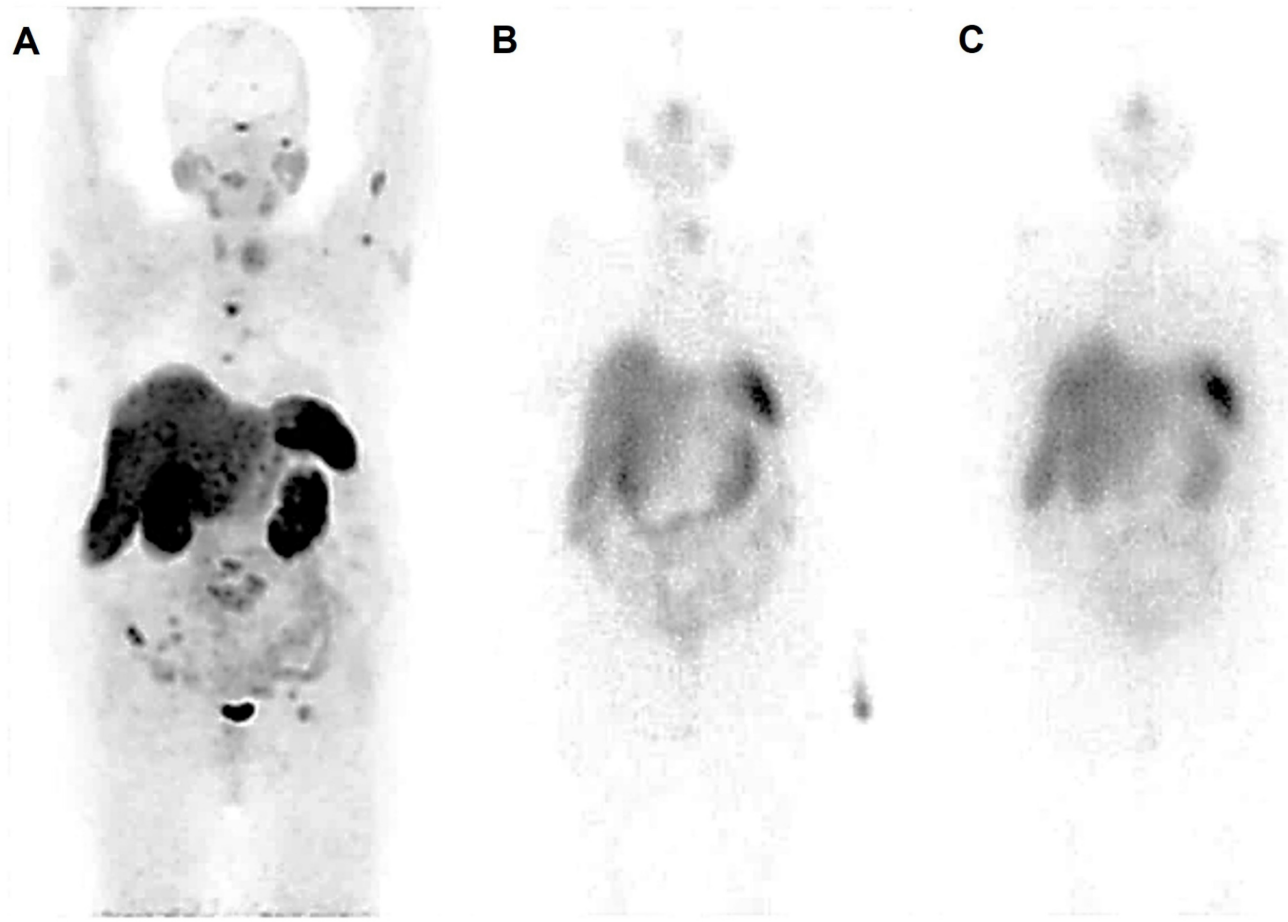
A 70-year-old female presenting with a history of medullary thyroid carcinoma (MTC), raised calcitonin and carcinoembryonic antigen (CEA) levels following thyroidectomy and documented metastatic lesions observed on anatomical imaging, underwent ^68^Ga-DOTATATE PET-CT to assess the feasibility of RLT, revealing tumoral lesions in the left thyroid lobe as well as pulmonary and multiple bone metastases (A). Hepatic lesions seemed to be non-functional, which could be the result of previous therapies. The patient underwent 2 cycles of PRRT with ^177^Lu-DOTATATE (cumulative activity, 14.8 GBq). Post-treatment scintigraphy after the first cycle (B) showed radiotracer uptake in the left thyroid lobe, right iliac wing and right sacrum. The second cycle (C) showed an accumulation of radiotracer in the left thyroid region. In follow-up, the levels of calcitonin and CEA increased, indicating progressive disease. Although the hepatic lesions appeared non-functional on the ^68^Ga-DOTATATE PET/CT scan, potentially due to prior treatments, the presence of multiple metastases throughout the body has been a significant factor in initiating ^177^Lu-DOTATATE therapy for this patient. Unfortunately, the patient died.

**Figure 3:**
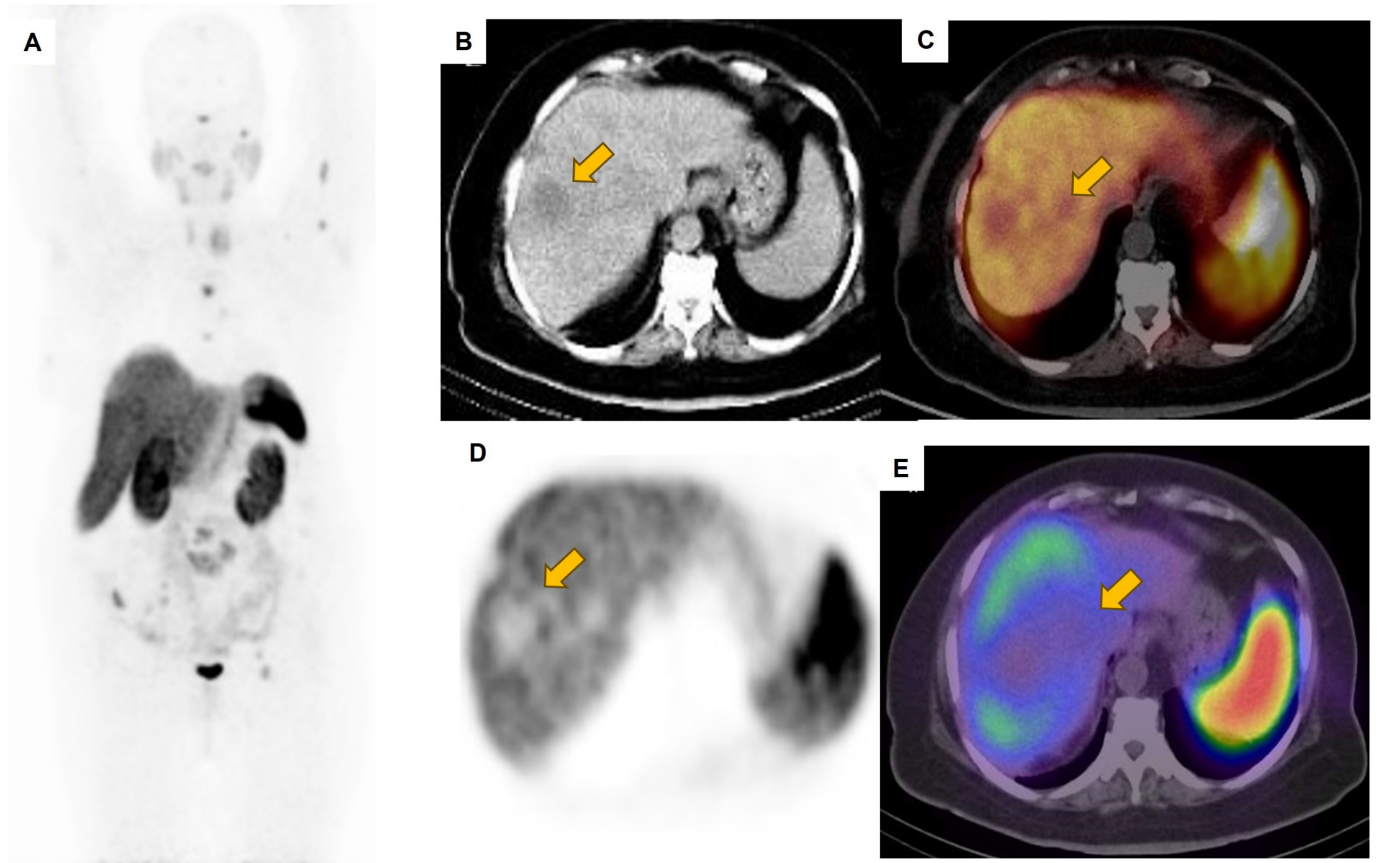
The maximum intensity projection was adjusted to a lower intensity to better highlight the hepatic lesions, which appeared non-functional (potentially due to prior therapies). The patient (same patient shown in Figure 2) subsequently underwent ^68^Ga-DOTATATE PET/CT (A). Transverse images from CT, PET, and PET/CT fusion are shown in panels B, C, and D, respectively. Non-functional hepatic lesions are further illustrated on the SPECT/CT image (E).

**Figure 4:**
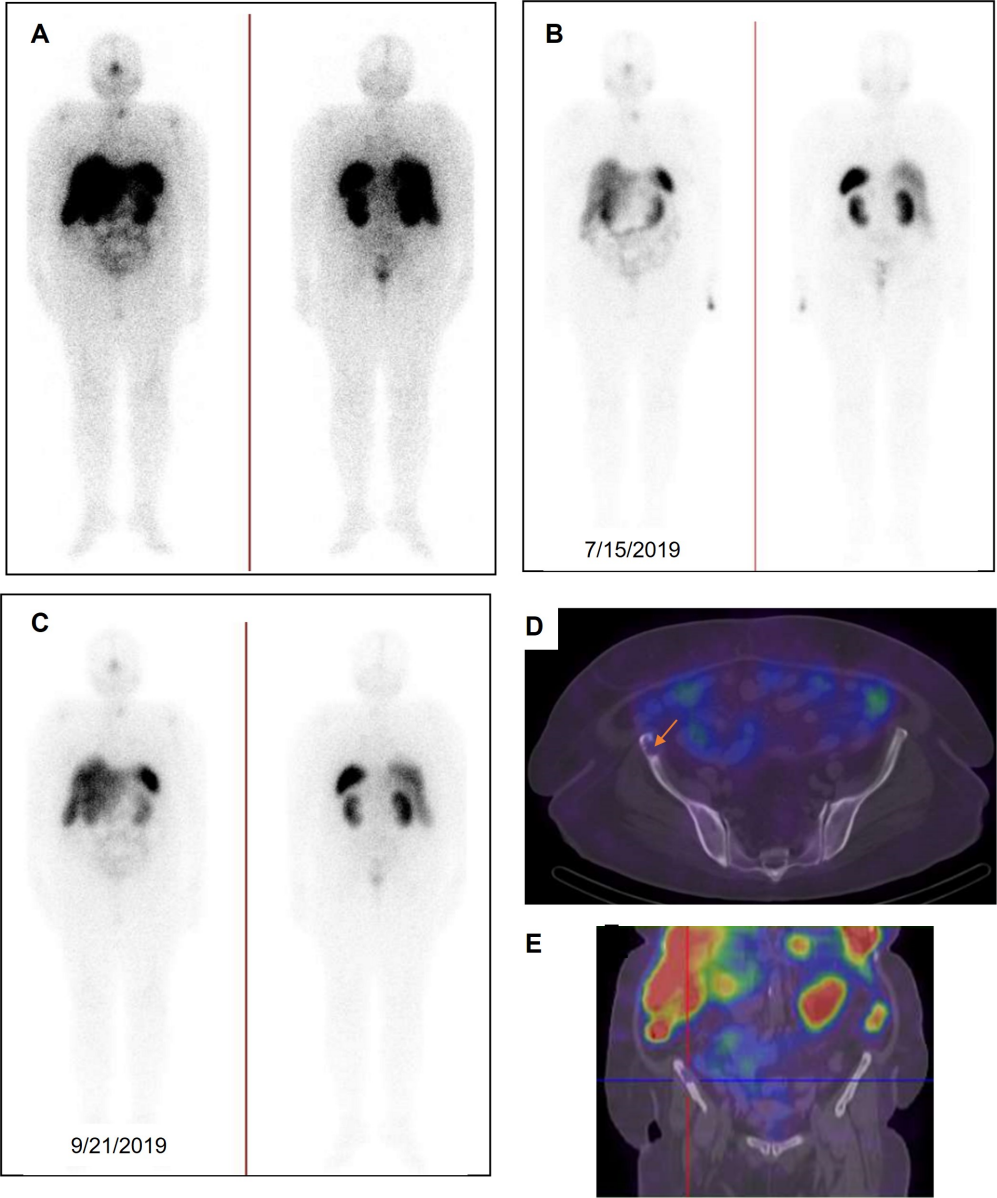
A) Displays higher intensity coloring, indicating a complete response to treatment in the right iliac wing and right sacrum after two cycles of PRRT with ^177^Lu-DOTATATE, as observed in the baseline PET image (same patient shown in Figures 2 and 3). B) Depicts planar images following the first treatment cycle, while C) represents those after the second cycle. D and E) Present SPECT/CT images to clarify the absence of uptake in the right iliac wing post-treatment, confirming the lack of residual disease activity. The lack of detectable bone lesions in post-treatment images may be due to their small size and the low spatial resolution of ^177^Lu SPECT scans. We believe that a single therapy cycle usually doesn't yield a therapeutic response, as there hasn't been enough time for the treatment to take effect; thus, these findings represent a baseline assessment at the start of the first cycle.

Tumors are known to display inherent diversity within patients and across different individuals, posing considerable obstacles for targeted cancer treatment 14. Using a comprehensive whole-body imaging technique is advantageous in this situation, as relying only on a single tumor biopsy may not capture the full diversity of the tumor. This can underestimate the genetic mutational burden, leading to treatment inefficacy or drug resistance 11, 14, 15. The field of theranostics, which has been in existence for more than seventy-five years, focuses on personalized therapy using molecular imaging. Within the domain of nuclear medicine physics (NMP), theranostics demands special attention. Moreover, the utilization of SPECT/CT greatly enhances the accuracy and sensitivity of whole-body scintigraphy by offering standardized uptake value (SUV) through meticulous sensitivity calibration. Consequently, this contributes to the progress of personalize medicine 14. Advancements in theranostics have recently been directed towards neuroendocrine tumors (NETs) by employing ^177^Lu-DOTA-peptides for therapeutic purposes, alongside ^68^Ga-DOTA-peptides as PET tracers for diagnostic imaging 15-17 (Figures 1-4).

**Figure 5:**
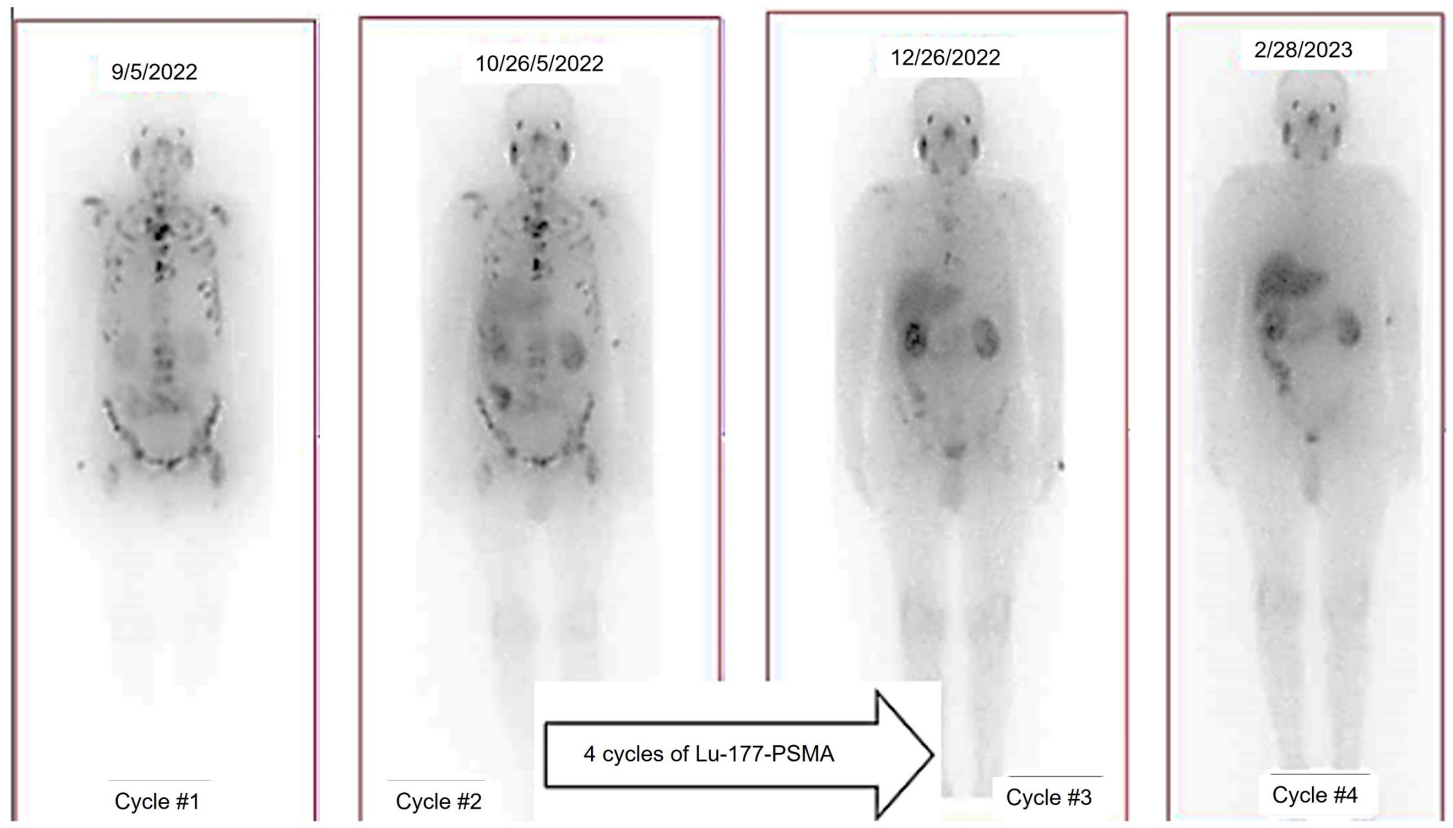
A 69-year-old man with history of radical prostatectomy + pelvic LND (initial PSA=32.0 ng/ml-Gleason score=4+4=8/10 with perineural and lymphovascular invasion). Owing to rising PSA level and widespread bone Mets in previous bone scan, the patient was referred to an oncologist for treatment planning. PSMA PET showed numerous PSMA-avid skeletal lesions throughout the skeleton involving the skull, skull base, sternum, ribs, spine, right scapula, both humeri, pelvic bones, proximal femora with SUV_max_ up to 43.7. Moreover, there are multiple PSMA-avid lymph nodes in the retroperitoneal and pelvic chains (para-aortic, aortocaval, left common iliac, left external iliac, and meso-sigmoid on the left side) with SUV_max_ up to 29.2. After three cycles, in comparison with the previous scans, excellent therapeutic response was noted and uptake in bony structures was reduced. Finally, after the 4^th^ cycle, there is radiotracer uptake in the salivary glands, lacrimal glands, kidneys, bladder and to some extent intestinal lumen as well as liver and spleen (no residual disease is noted; PSA=0.5).

In addition, the utilization of ^177^Lu-based SPECT/CT imaging offers several advantages including radiation dosimetry assessment, evaluation of treatment efficacy, and minimizing inadvertent radiation exposure to critical organs such as the kidney, bladder, and red marrow. Specifically for metastatic prostate cancer (mPC), there have been advancements in the development of diagnostic ligands like the ^68^Ga-PSMA ligands for PET imaging and therapeutic ligands such as ^177^Lu-PSMA-617 15, 18 (Figure 5).

## Radionuclides

### Alpha vs. Beta particles

#### Variations in Linear Energy Transfer (LET) and Relative Biological Effectiveness (RBE)

**Figure 6:**
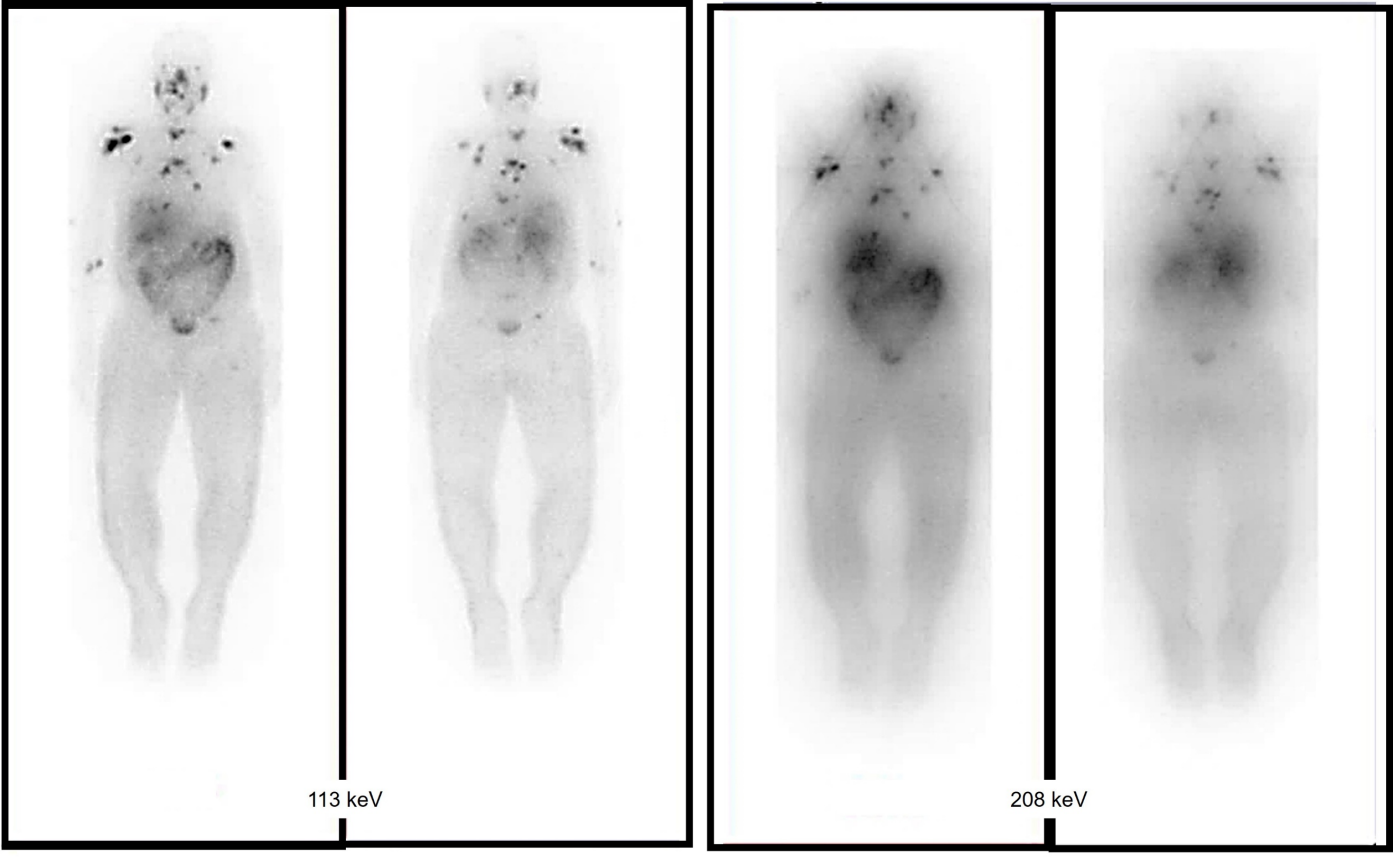
Comparing the effect of choosing the best energy window with using low-energy high resolution (LEHR). A 60-year-old male with known case of prostate adenocarcinoma. Widespread bone metastasis throughout the spine (C6, T1, T8, T9, T10, T11, sacrum), sternum, right femoral head, right clavicle, both scapulae and lateral aspect ribs bilaterally. Acquisitions of these two peaks (113 and 208 keV) using a low-energy high resolution (LEHR) collimator, with the 113 keV peak being regarded as the primary peak, and employing a 20% energy window.

The biological effects of radiation are largely mediated through its interaction with DNA. In particular, alpha particles, due to their high LET, induce dense ionization tracks leading to complex DNA damage, while beta particles, with lower LET, tend to produce more sparse ionization patterns. This difference in ionization density results in distinct patterns of DNA damage and repair mechanisms*.* β-particles have a low LET of around 0.2 keV/µm and can travel a significant distance of approximately 2-12 mm (equivalent to 20 to 120 cell lengths) 19-21. The clinically used β-emitting radionuclides, ¹⁷⁷Lu and ⁹⁰Y, are ordered in increasing magnitude according to their maximum energy emission and path length 22. β-energy of ^177^Lu is 0.5 MeV along with two primary gamma-energies of 113 and 208 keV, with respective yields of 6.1% and 10.3%. These gamma emissions facilitate imaging and assessment of radiotracer distribution within the body. Due to scatter (with lower energy) from the 208 keV peak into the 113 keV window, the more intense energy peak was exclusively selected. At the first author's facility, the 113 keV and 208 keV peaks were acquired using a low-energy high-resolution (LEHR) collimator. The 113 keV peak was selected as the primary peak, with a 20% energy window applied, as shown in Figure 6. However, imaging protocols can vary across facilities, depending on equipment, collimators, and energy window settings. For instance, one facility may use a LEHR collimator with a 20% energy window around the 113 keV peak, while others may prioritize the 208 keV peak, employ different collimators, or adjust energy window settings to optimize spectral capture and minimize noise or interference.

**Figure 7:**
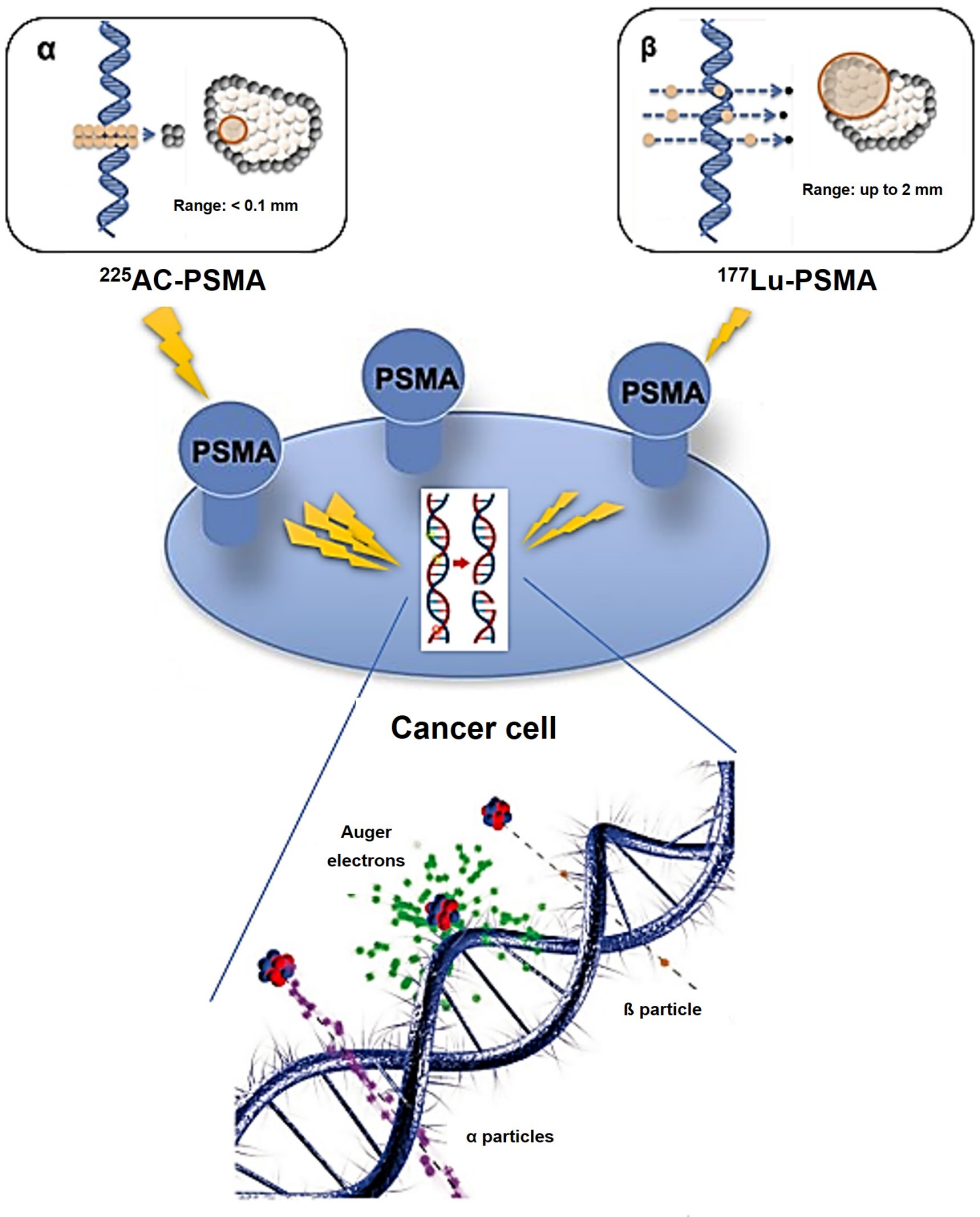
Schematic illustration of the effects of Alpha/Beta particles and their linear energy transfer (LET).

Conversely, an α particle bears a positive charge and is approximately three orders of magnitude larger than a β particle. As a result, α particles exhibit a notably higher LET of 80 keV/µm in contrast to β particles and cover a considerably shorter distance, usually falling within the span of 50-100 µm (equivalent to one to three cell lengths) as illustrated in Figure 7 20.

Theoretically, Auger electron can be used to deliver biological effects to cells. Auger electrons are electrons with low energy that are emitted by radionuclides as a result of decay through electron capture, leading to the ejection of an electron from an electron orbit surrounding the nucleus. These electrons have a restricted travel distance, usually falling within the range of nanometers to micrometers. Similar to α particles, Auger electrons have a high LET and a short range, which enables them to cause localized DNA damage. Despite the limited clinical application of Auger emitters at present, research involving animals and patients treated with Auger emitters has shown promising outcomes, indicating the need for further exploration 23, 24. In the study conducted by Al-Ibraheem *et al.*, Terbium-161 (^161^Tb) has emerged as a promising radionuclide for radiotheranostics in different cancer types, such as metastatic castration-resistant prostate cancer (mCRPC). The researchers delve into the potential of ^161^Tb as an exemplar of auger emission and its ability to rival the effectiveness of ^177^Lu 25. Relative biological effectiveness (RBE) distinguishes the biological effects of different radiation types beyond LET. Different types of radiation, like α particles and photons, can result in varying levels of biological damage at the same dose. RBE is calculated as the ratio of the dose required from a standard radiation source to produce an equivalent biological effect to that of test radiation. Factors like dose, dose rate, cell radiosensitivity, repair capabilities, and LET influence RBE. α particles and high LET radiation have a high RBE, indicating they are more effective at causing biological damage compared to β particles or γ emissions (Table 1).

**Figure 8:**
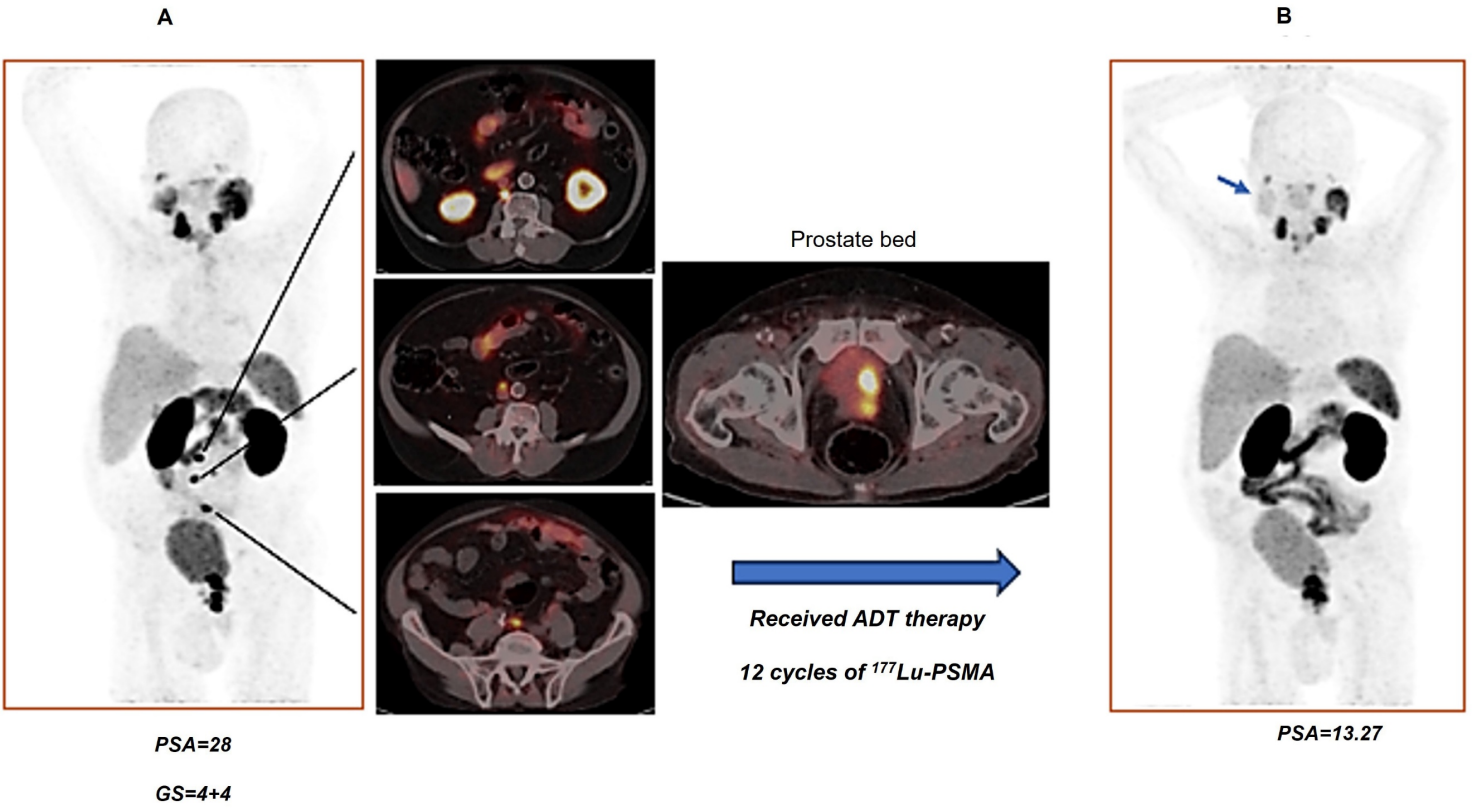
A (87 years old man with history of prostate adenocarcinoma, underwent hormonal therapy (ADT), referred for recurrence evaluation. A) There are multiple lymph nodes with PSMA uptake in the retrocaval at level L2; (SUV_max_=40.87), precaval (SUV _max_=23.59), paraaortic (SUV_max_=9.20), aortic bifurcation (SUV max=24.22) as well as mild PSMA uptake in the left internal iliac lymph node. The prostate gland showed PSMA uptake (SUV_max_=42.12) with invasion to bladder. B) received ADT therapy and 12 cycles of ^177^Lu-PSMA up to 2 months before last PSMA PET imaging. Right parotid gland showed decreased physiologic tracer uptake comparing with left parotid gland (because of ^177^Lu-PSMA therapy- blue arrow in the right image). All retroperitoneal and paraaortic LNs which is mentioned in previous scan showed no more uptake. Intense increased PSMA uptake in the prostate gland, involving a significant portion of the gland, with extension to the seminal vesicles and invasion of the posterior wall of the urinary bladder. Comparison with the previous scan showed progression. Findings are consistent with partial response to therapy.

Understanding RBE is crucial for assessing risks and developing radiation protection strategies 26. The biological outcome of a radiation dose is influenced by the rate at which it is administered. When lower dose rates are used, there is a greater opportunity for repair of sublethal DNA damage compared to high-dose-rate delivery. In contrast to conventional external-beam radiation therapy (EBRT), which delivers a high level of radiation in a short period of time, most RLTs are administered at lower dose rates that decrease exponentially over time. This has important clinical implications for the established toxicity limits for organs at risk (OARs) such as the kidneys, liver, and salivary glands (Figure 8).

The current dose limits are primarily based on data from EBRT, and further research is necessary to determine whether higher doses can be tolerated due to the prolonged delivery of radiation in RLTs 3, 27.

#### Distribution/retention/effective half-Life and administered activity

**Figure 9:**
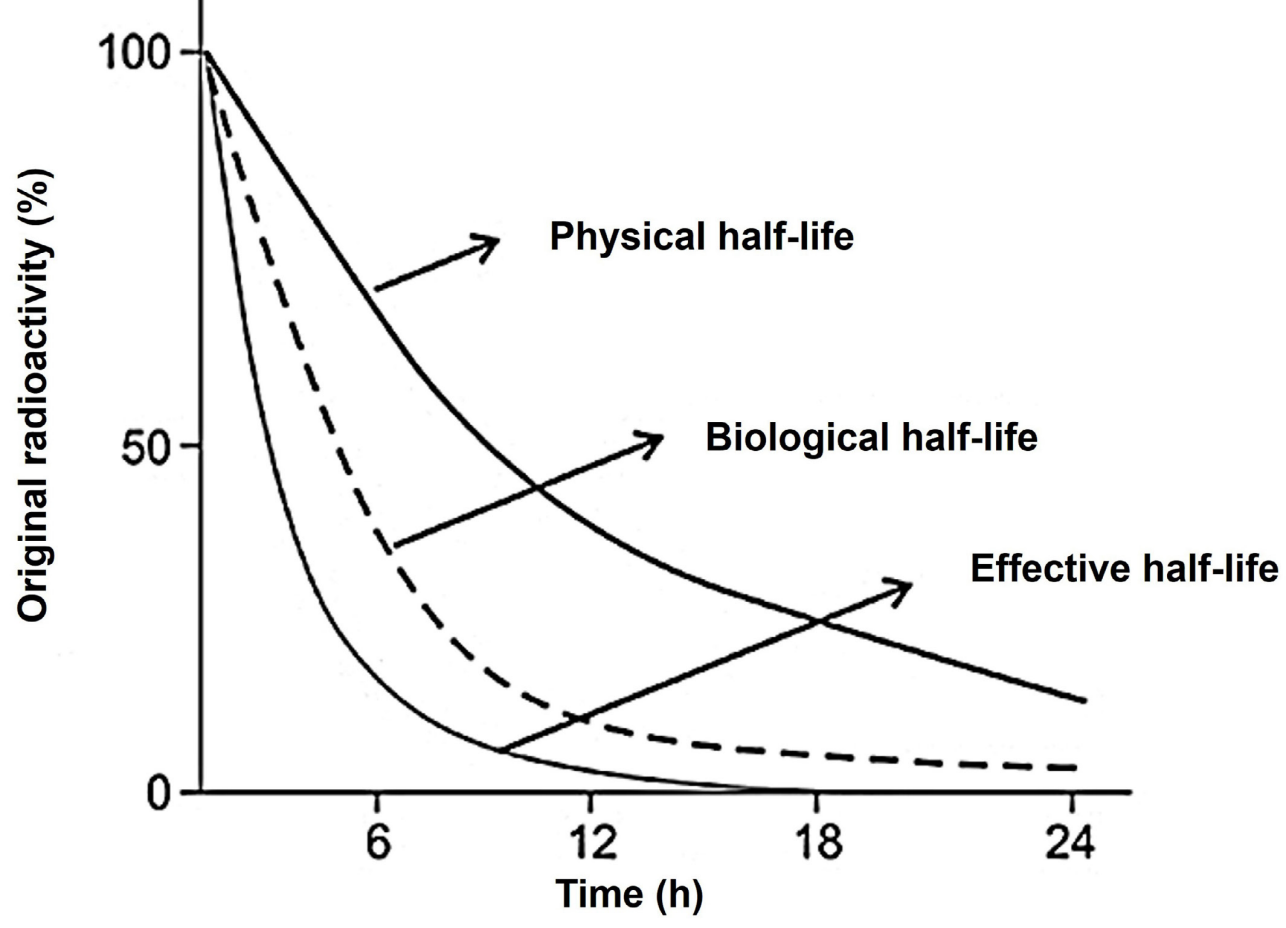
The effective half-life, accounting for both physical and biologic decay, is the time for half of the radiotracer activity to clear from the body and is consequently shorter than both the biologic and physical half-lives.

The duration for a radionuclide to decay to half of its initial activity is known as the physical half-life. On the other hand, the biologic half-life refers to the time it takes to eliminate half of the administered agent through biologic clearance alone. The effective half-life represents the time required for half of the radiotracer activity to clear from the body, accounting for both physical and biological decay. It is important to note that the effective half-life is shorter than both the biologic and physical half-lives due to the combined effects of both decay processes (Figure 9).

Prolonged presence of a substance in a particular tissue is associated with a higher probability of causing harm to that tissue, whether it is a malignant growth or a healthy organ. For example, low extraction fraction of a radiotracer in the bloodstream raises the chances of radiation-induced damage to the bone marrow. A slower elimination rate, leading to an extended biological half-life, increases the risk of bone marrow toxicity from the same dosage administered 28, 29. Administering a higher activity level can potentially improve the therapeutic impact on specific lesions, but it also increases the potential harm to organs at risk and non-target tissues. In cases where significant toxicity occurs, it is prudent to consider temporarily or permanently discontinuing the treatment, depending on the severity of the toxicity, to allow for the recovery of organ function. If the patient's organ function improves and they are able to resume treatment, it is generally advised to administer a reduced dose in subsequent cycles to minimize the chances of recurring toxicity. The protocols for managing side effects differ depending on the specific type of radiotherapy treatment being utilized 30, 31. The distribution and uptake of the therapeutic agent in tissues contribute to tissue damage, impacting both the antitumor therapeutic effect and organ toxicity. Targeted treatment requires tumor expression of the target (Table 2), and a high level of uptake in tumors is indicative of a more substantial therapeutic response 32-35.

## Radionuclide Therapy

### Current Clinical Practice and Future Directions

#### Somatostatin Receptor-targeted Radionuclide Therapy

NETs in the pancreas, lung and midgut that exhibit somatostatin receptors (SSRs) expression, in particular the subtype 2 (SSR2a), can be identified through the utilization of ^68^Ga-DOTA peptides such as DOTATATE, DOTANOC, and DOTATOC. A theranostic strategy involving ^177^Lu-DOTA-peptides or ^90^Y-octreotate can be employed for the treatment of these tumors 37. During the critical third phase of the NETTER-1 trial, individuals diagnosed with advanced midgut NETs who received treatment with ^177^Lu-DOTATATE experienced a notable rise in progression-free survival (PFS) after 20 months in contrast to the control cohort (65.2% versus 10.8%, respectively). Furthermore, a significantly increased tumor response rate was noted 38. The NETTER-1 trial, unfortunately, did not show a statistically significant improvement in overall survival (OS) after 5 years. This lack of significance can be attributed in part to a substantial crossover rate within the control group, with 36% of participants eventually undergoing SSR-coupled radionuclide therapy 39. Consequently, significant clinical guidelines integrated this treatment into their protocols. Moreover, the NETTER-1 study illustrated that patients treated with ^177^Lu-DOTATATE encountered a superior quality of life across various domains such as overall health, body image, functionality (both general and occupational), diarrhea, pain, fatigue, and concern about the illness. Further investigation and advancement in the field of RLT is imperative for patients diagnosed with advanced midgut NETs. A recent phase 2 clinical trial focused on exploring the efficacy of an SSR-targeted α-particle therapy utilizing ^225^Ac-DOTATATE 40. The results of this trial showcased promising responses and PFS outcomes specifically for gastroenteropancreatic neuroendocrine tumors (GEP-NET). Previous preclinical and initial clinical research has suggested that SSR2 antagonists may exhibit a higher receptor binding density, leading to a more favorable tumor-to-background ratio (TBR) and improved lesion detection when compared to agonists 41, 42. The heightened receptor binding capacity of antagonists has the potential to increase sensitivity, enabling the treatment of tumors with lower receptor density. Table 3 presents a comprehensive overview of five ongoing trials that employ investigational theranostic pairs based on SSR antagonists for SSR-expressing NETs and meningiomas.

This highlights the active and innovative nature of RLT in this field. Furthermore, there is a growing interest in investigating the use of SSR-coupled RLT at an earlier stage in the treatment process. This is exemplified by the NETTER-2 trial, which aims to evaluate the efficacy of ^177^Lu-DOTATATE as a first-line treatment for untreated patients with metastatic grade 2-3 NETs. The trial involves randomization of patients to receive either ^177^Lu-DOTATATE in combination with long-acting octreotide or high-dose long-acting octreotide alone.

#### Prostate-specific Membrane Antigen-targeted Radionuclide Therapy

**Figure 11:**
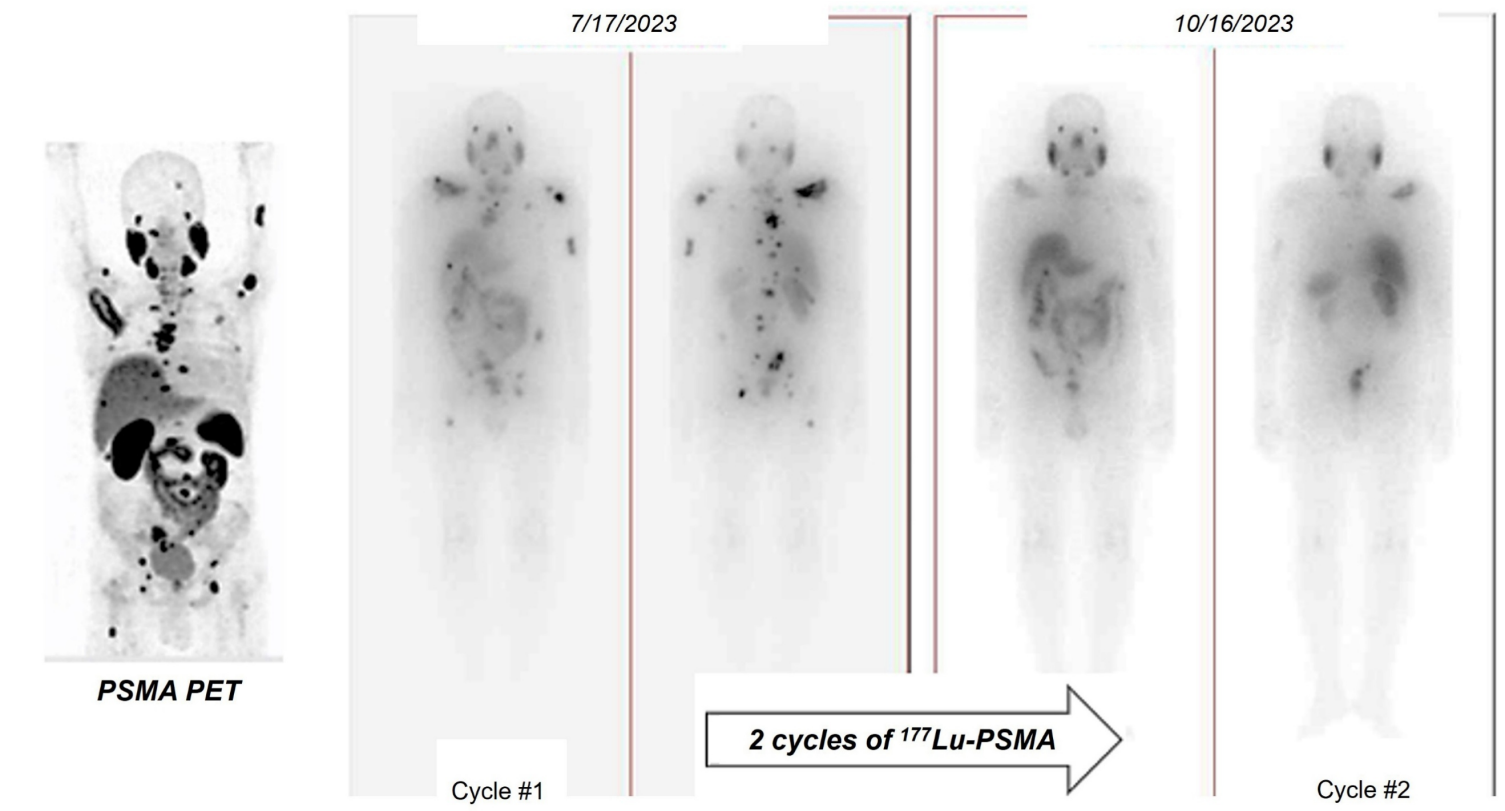
A 63-year-old man with history of prostate adenocarcinoma (GS=4+3=7/10 and bone mets) underwent the chemotherapy and Abiratrone with increasing the PSA level to 135 ng/ml. The scan showed increased tracer uptake in the right scapula, right iliac and sacrum. Physiologic tracer uptake was seen throughout salivary gland, liver, GI tract and urinary bladder. Metastatic involvement in the right scapula, right iliac and sacrum which is comparing with previous scan showed partial response to treatment.

In the TheraP trial's second phase, the utilization of ^177^Lu-PSMA-617 demonstrated a more remarkable decrease in prostate-specific antigen (PSA) levels when compared to cabazitaxel for patients with metastatic castration-resistant prostate cancer (mCRPC) that was progressing 43. The phase 3 VISION trial (ClinicalTrials.gov no. NCT03511664) revealed additional results indicating that individuals who underwent treatment with ^177^Lu-PSMA-617 demonstrated improved PFS as observed through imaging, as well as a greater median OS (15.3 vs. 11.3 months, respectively) in comparison to patients who solely received standard-of-care treatment 36. The FDA has recently approved ^177^Lu-PSMA-617 for the treatment of mCRPC, in addition to the utilization of ^68^Ga-PSMA-11 PET for diagnostic imaging purposes (Figure 11).

^18^F-DCFPyL PET has been utilized in clinical trials as the diagnostic pair targeting PSMA 44, 45. The realm of PSMA-coupled RLT is currently experiencing significant clinical advancements, concentrating on the advancement of targeted α-particle therapy in conjunction with PSMA ligands. A groundbreaking study in 2016 showcased an impressive treatment outcome through the utilization of ^225^Ac-labeled PSMA ligands (^225^Ac-PSMA-617) in mCRPC patients 46. Additional research has provided further evidence of the effectiveness and safety of ^225^Ac-PSMA-617 as a viable treatment choice. A comprehensive analysis and evaluation of 256 patients who underwent treatment with ^225^Ac-PSMA-617 revealed an impressive overall biochemical response rate of 62.8%. Furthermore, a molecular response rate of 74% was observed through the use of ^68^Ga-PSMA-11 PET/CT scans. The median estimates for PFS and OS were determined to be 9.1 months and 12.8 months, respectively 47. Additional randomized controlled trials in the future are essential to further establish the effectiveness of therapy and the benefits it provides for survival. Currently, phase 1 and 2 trials, as well as registry data, are actively enrolling patients. Despite the increased risk of xerostomia, ^225^Ac is actively being studied in prospective clinical trials. An ongoing phase 2/3 trial (NCT06402331) is underway, with additional trials anticipated to commence soon, potentially paving the way for future FDA approval.

The potential application of this approach for eliminating the primary tumor site is currently being investigated in clinical studies, including the recently completed LuTectomy trial (ClinicalTrials.gov registration no. NCT04430192). These trials will provide valuable insights into the feasibility of utilizing targeted treatment in earlier stages of the disease.

To provide a robust foundation for current clinical practices, this section included an expanded discussion on recent clinical trials. Emphasis has been placed on methodological nuances, such as trial design, patient selection criteria, and outcome evaluation. Detailed case studies and data analyses have been incorporated to illustrate these points, with further reading provided in 48.

## Biology and DNA damage

Recent advances in radiobiology have significantly enhanced our understanding of the molecular and cellular responses to radiation. In this section, we elaborate on key mechanisms, such as DNA damage response, cell cycle checkpoints, and repair pathways. For further detailed insights, readers are encouraged to consult the extensive reviews cited herein 49, 50.

### Cellular Repair Mechanisms

**Figure 12:**
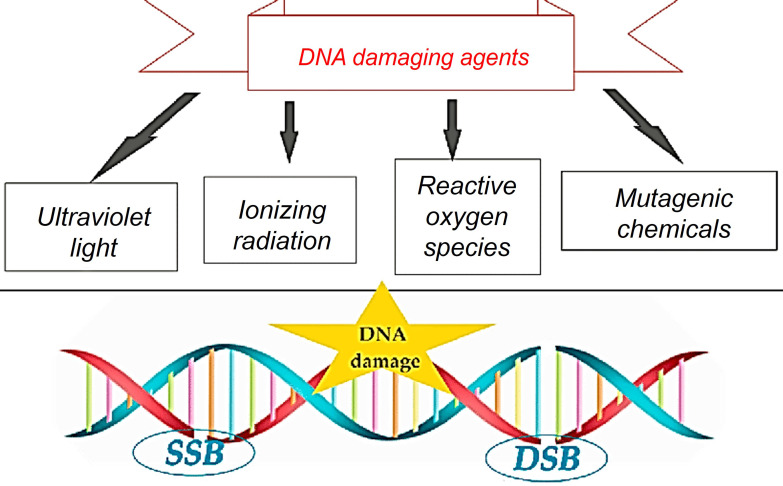
DNA damage is caused by external factors such as environmental, physical, and chemical agents like ultraviolet and ionizing radiation, alkylating agents, and crosslinking agents.

DNA is a pivotal molecule that preserves essential genetic information necessary for the growth and survival of organisms. It is renowned for its relatively superior stability when compared to other biological compounds 51, 52. Although DNA is stable, it can be modified by internal and external factors, leading to harmful mutations in cells 53. Various factors, like replication errors, mismatched DNA bases, spontaneous deamination, and oxidative damage from reactive oxygen species (ROS), can cause DNA damage 54, 55. DNA damage can result from external factors like UV radiation, chemicals, and other environmental elements 52, 56 (Figure 12).

**Figure 13:**
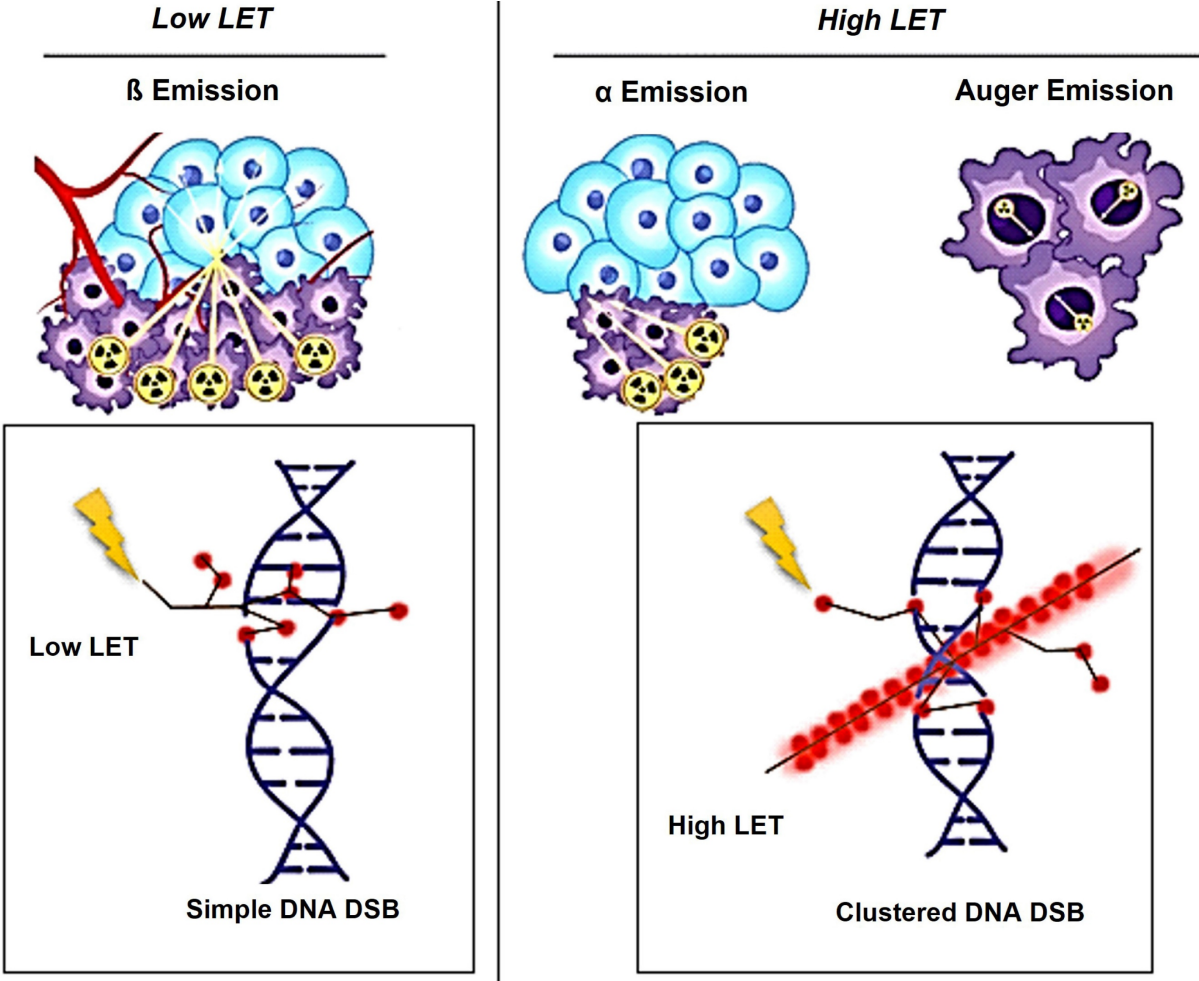
These external factors can lead to DNA single-strand breaks (SSB) or double-strand breaks (DSB), where SSB involves the breakage of one DNA strand while the other remains intact, and DSB involves the breakage of both DNA strands.

External factors can cause DNA single-strand breaks (SSB) or double-strand breaks (DSB). SSB is when one DNA strand breaks, while DSB is when both strands break 57 (Figure 13). In cancer, disruptions in DNA damage response can induce mutations and instability, driving disease progression 53, 58, 59. Tumors grow quickly due to unstable genes, which can be treated with chemotherapy, radiation therapy, or radiopharmaceuticals 53, 60. It is crucial to understand the effects of targeted radiation therapy on tumors and minimize unintended harm by studying DNA damage and repair mechanisms triggered by different radioisotopes and molecular targets 56, 59.

### High-LET particles and its effects on DNA damage

*Alpha-particle emitters.* The use of α-particle emitters in targeted therapeutics is gaining attention due to their high energy levels (4-8 MeV) and limited emission range in tissues. Using a targeted radiopharmaceutical labeled with an α-emitter allows for precise delivery of radiation to tumors while minimizing exposure to healthy tissues 61-63. Research suggests that α-emitting radioisotopes, with their high energy and charge, can directly interact with DNA, leading to the formation of irreversible DSBs within the DNA structure 64. α-emitters cause direct DNA damage and can also generate free radicals, leading to some indirect DNA damage 65. The distinction between direct and indirect DNA damage and their long-term clinical effects is unclear and requires further investigation in future research.

### Low-LET particles and its effects on DNA damage

*Beta-particle emitters.* The main connection between β-emitters' toxicity in mammalian cells and their effects is the indirect DNA damage caused by ROS and oxidative stress 66, 67. These mechanisms primarily create SSBs, with DSBs less commonly found in affected cells 59, 68. Research shows that β-emitters, such as ^177^Lu, primarily work by creating SSBs for therapeutic purposes. While tumor cells can repair these breaks, the accumulation of damage can overpower repair processes, leading to cell death and tumor reduction 69. However, the use of ^177^Lu-DOTATATE or ^177^Lu-PSMA ligands 70, 71 has led to an increase in DSBs with higher doses. Research has been done to study how ^177^Lu, a common β-emitter, affects cellular death mechanisms 72-74. Beta-emitters moving through biological tissue primarily cause DNA damage indirectly by generating free radicals like ROS, leading to chemical reactions. These processes can result in early or late apoptosis, mutations, and genomic instability 75. β-particles can cause direct DNA damage by interacting with DNA, displacing electrons and causing ionization. This can lead to SSBs and potentially reversible DSBs 76, 77.

### DNA damage repair pathways in the cellular response to Beta/Alpha particle emitters

Cancer cells lack DNA repair pathways, making them susceptible to radiotherapy and DNA-damaging substances. EBRT and RLT exploit this vulnerability, causing irreparable DNA damage and leading to the death of cancer cells 56, 59. An investigation found a link between DNA damage response irregularities and increased PSMA expression in prostate cancer patients, potentially improving response to PSMA-targeted RLT 78. In the context of ^225^Ac-PSMA-617 treatment, two individuals with mCRPC and BRCA1 gene mutations showed longer survival compared to patients without DNA damage response (DDR) mutations 79. Radioimmunotherapy using α-emitters is less likely to cause resistance in cancer cells compared to beta-emitters, possibly due to the creation of permanent double-strand breaks in DNA 80, 81. However, resistance to α-emitters can be attributed to various DDR mechanisms and signaling pathways within cancer cells 82-84. The selection of a repair pathway for DNA DSBs is complex and influenced by factors, such as the number and type of DSBs 81.

### Impact of dose rate on DNA damage and repair

Dosimetry in RPT lacks a specific definition found in established EBRT protocols, making it difficult to accurately calculate and understand absorbed doses and their dispersion in specific tissues or organs 85. Determining the dose rate (DR) is crucial for understanding radiation's impact on cellular structures, gene expression, cellular responses, and cell death mechanisms. Radiopharmaceuticals release radiation gradually, resulting in a fluctuating and declining dose rate. The energy and distribution of the dose depend on the radionuclide used. Factors like physical half-life, specific activity, biological half-life, and cell repair ability influence the DR in radiation therapy. Lower dose rates cause less harm and dispersed radiation, leading to reparable sublethal damage 85, 86. Researchers have found that repair foci increase non-linearly at low dose rates, suggesting repair mechanisms may be more effective at low doses than high doses 87. Contradictory findings challenge the idea that low levels of radiation do not activate genes necessary for DNA repair and only result in minimal or no repair of DNA damage 88, 89. Studies suggest that DNA repair is limited at low dose rates due to insufficient DNA damage initiation, leading to inadequate activation of repair genes. This disparity in cellular response to double-strand breaks is evident when comparing low and high doses of low LET radiation 88, 90-92. Lower doses may increase the risk of cancer more than estimated based on higher doses using linear extrapolation due to the absence of repair mechanisms 89, 93. The bystander effect shows how indirect harm can result from exposure to RLT 94, 95. The spread of damage from irradiated cells to nearby non-irradiated cells is significant 96, 97. Therefore, it is reasonable to suggest that bystander signaling may have been less effectively induced at the higher dose rate compared to the lower dose rate. Understanding the effects of different radioisotopes on DNA damage and repair processes, estimating absorbed doses through dosimetry, comparing radioisotopes with varying energy and emission profiles, and evaluating photonics versus electronic emissions are all crucial for advancing RLT. Enhancing preclinical models, studying RLT effects on the tumor microenvironment, exploring combination treatments, and assessing short and long-term toxicities are also essential. Collaboration among oncologists, nuclear medicine specialists, radiation oncologists, physicists, and biologists is necessary to bridge this knowledge gap.

## Dosimetry

### Investigational uses of dosimetry in radionuclide therapy

In RLT, individual variations in peptide pharmacokinetics among patients necessitate personalized treatment strategies, such as adjusting the number of treatment cycles or the amount of administered activity 98. One approach to therapy planning involves determining the maximum tolerable absorbed dose to non-target organs, known as the "as high as safely attainable" (AHASA) approach 99, as opposed to the traditional "as low as reasonably achievable" (ALARA) approach, which aims to minimize radiation exposure to non-target tissues. Despite this, the current practice typically involves administering a fixed activity of 7.4 GBq per cycle, as seen in the NETTER-1 trial. As a result, personalized dosimetry is often conducted primarily to ensure safety, and assess the absorbed dose to the tumor, rather than to optimize the administered activity, and evaluate the dose-response relationship. Accurate estimation of the activity in the targeted organs at multiple time points is essential for patient dosimetry 100, 101. Hence, the preliminary calibration and quantification steps play a crucial role 102, 103. Despite the existence of Medical Internal Radiation Dose (MIRD) guidelines 104, 105, there is a pressing need for a standardized dosimetry protocol to assess safety, and toxicity, and conduct dosimetric evaluations. According to a recent review by Huizing *et al.* 106, dosimetry in RLT is not commonly practiced due to various challenges in implementation, the time-consuming nature of non-standard dosimetry methods, and the lack of supporting evidence in the literature.

To overcome these issues, commercial software programs have been created to address challenges in phantom and patient-specific dosimetry (Tables 4 and 5). These programs offer advanced tools for precise radiation dose calculations and analysis, catering to medical physicists, radiation oncologists, and other healthcare professionals involved in radiation therapy planning. Phantom dosimetry involves measuring and computing radiation doses using phantoms that mimic human tissues and organs 107. These phantoms validate RLT plans and delivery systems, providing features for phantom design, dose calculation, and analysis. Patient-specific dosimetry tailors RLT plans to individual patient characteristics, offering tools for precise dose calculation and optimization based on factors like patient anatomy and tumor location 103. These programs use sophisticated algorithms to simulate radiation interaction with patient tissues, resulting in accurate dose distribution calculations.

The significance of ^177^Lu-based SPECT/CT in internal dosimetry for NETs has surpassed that of traditional diagnostic PET or SPECT imaging. This is mainly attributed to its direct connection to therapeutics within theranostics. Unlike standard dosimetry, which necessitates the identification of organs throughout the body and multiple sequential whole-body PET scans 108, this approach can pose challenges in routine theranostics practice due to the considerably longer half-lives of therapeutic radioisotopes such as ^177^Lu compared to ^18^F. A recent study has demonstrated that a dosimetry technique utilizing single-time point (STP) imaging exhibits reduced variability when compared to more intricate methods, such as employing multiple time points (MTP) for exponential fitting to determine residence time. Even though STP dosimetry is still under investigation, some studies have demonstrated that a dosimetry technique utilizing STP imaging could reduce variability when compared to more intricate methods, such as employing MTPs for exponential fitting to determine residence time. When dosimetry is performed with STP imaging instead of MTP imaging, a wider safety margin can be used for the kidney-absorbed dose limit. Protocols can also be designed to switch to MTP imaging in the next cycle as a cautionary measure only for those patients whose STP-estimated kidney- absorbed dose in the previous cycle is above a limit predetermined on the basis of the predicted error distribution for the specific STP model 103, 107, 109-111.

**Figure 14:**
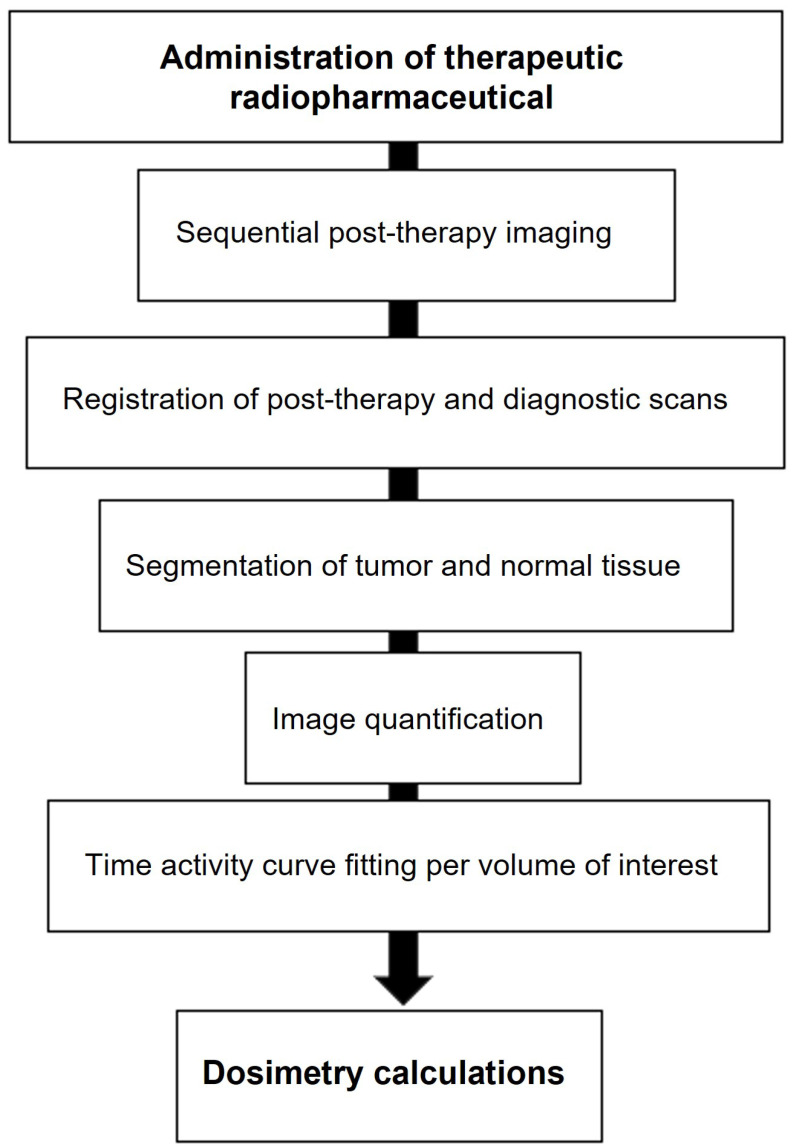
Schematic workflow for clinical dosimetry in RLT.

Given the strong correlation between internal dosimetry and the estimation of external radiation exposure, dosimetry plays a crucial role in Nuclear Medicine Physics (NMP) within the field of theranostics. Particularly, dosimetry holds significant promise in the investigation of ^177^Lu-conjugated radiopharmaceuticals in clinical practice (Figure 14).

The emission of β-particles by ^177^Lu serves to target and eliminate cancer cells, while the emission of γ-photons aids in imaging purposes. Research indicates that utilizing planar whole-body γ-camera protocols and SPECT/CT for multiple-time-point imaging of ^177^Lu-DOTATATE enables quantitative evaluations of the radionuclide's distribution within individual patients over time. This approach facilitates personalized dosimetry calculations for both tumors and critical organs, such as the kidney. Theoretically, comprehending the absorbed dose could assist in adjusting the activity in subsequent treatment cycles appropriately and predicting outcomes. Furthermore, with the advancement of Deep Learning (DL) techniques, there exists the potential for automatically delineating target lesions and critical organs in this particular context.

#### Molecular imaging for dosimetry

The accumulation of a particular radiopharmaceutical is contingent upon the intensity and variability of target expression in both the tumor and normal organs, in addition to the biokinetic profile of the radiopharmaceutical 103. Molecular imaging is essential for evaluating the biodistribution of radiopharmaceutical activity in individual patients undergoing treatment. Various imaging techniques, including SPECT, planar imaging, and hybrid imaging, can be utilized to assess the distribution of therapeutic radiopharmaceuticals. However, quantitative SPECT/CT is considered the optimal method for precise quantitative measurements 105. The appropriate timing for imaging will be determined by the absorption and elimination patterns of the particular radiopharmaceutical agent. However, it is customary to acquire at least three SPECT time points following the administration of the treatment 104, 112. These images serve the purpose of delineating specific areas of interest, where the organs at risk are accurately identified concerning the therapy being administered. By capturing a momentary snapshot of the concentration and distribution of the radiopharmaceutical, these images enable the creation of time-activity curves (TACs) for each region of interest (ROI). These curves provide a visual representation of how the activity accumulates, spreads, and is eliminated by a particular region throughout treatment. The area under the curve reflects the total accumulated activity, which, when multiplied by the energy released per decay and the absorbed fraction specific to the source and target, determines the absorbed dose. This absorbed dose per cumulated activity is known as an S factor, which represents a radiation transport factor unique to the source and target. In the case of normal organs, these factors are often computed through particle transport simulations and are commonly employed for estimating absorbed doses and assessing risks at a population level.

#### Voxel-based dosimetry

**Figure 15:**
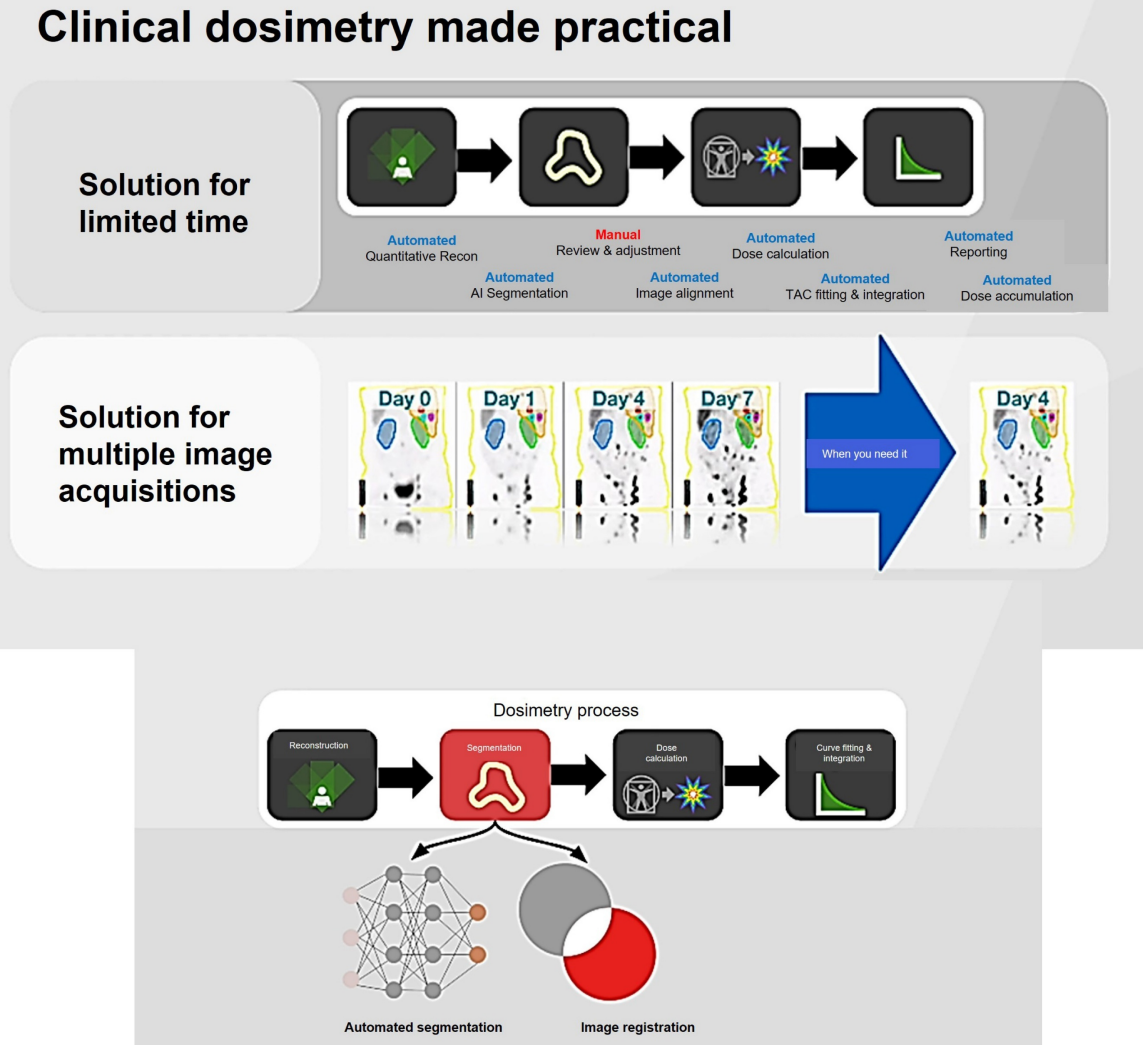
Voxel-wise dosimetry process (MiM dosimetry software^'^s workflow).

Organ-level dosimetry is crucial in the estimation of dose absorption, under the assumption of a uniform distribution of activity within organs and tumors. Conversely, sub-organ-level dosimetry, particularly at the voxel scale, takes into account patient-specific nonuniform activity distributions within organs and tumors. Voxel-wise dosimetry requires the calculation of energy deposition by transforming the three-dimensional activity distribution into a dose distribution through the utilization of data obtained from particle simulations (Figure 15).

This strategy represents the varied distribution of radiopharmaceuticals found in organs and tumors. Most methods for calculating voxel dose can be divided into four categories:

- The first and simplest way to calculate absorbed dose is that all doses are assumed to be: The associated radiation is absorbed locally, increasing the total decay number (TIAC).
- The second calculation method is based on the dose point kernel (DPK). TIAC images can be converted into dose distribution by collapsing the image in DPK. Another drawback is that DPK is dependent on voxel size. The DPK method cannot be considered as it is limited to one material.
- The third method is Monte Carlo simulation (MC).
- The fourth method is internal dosimetry using deep learning.

**Figure 16:**
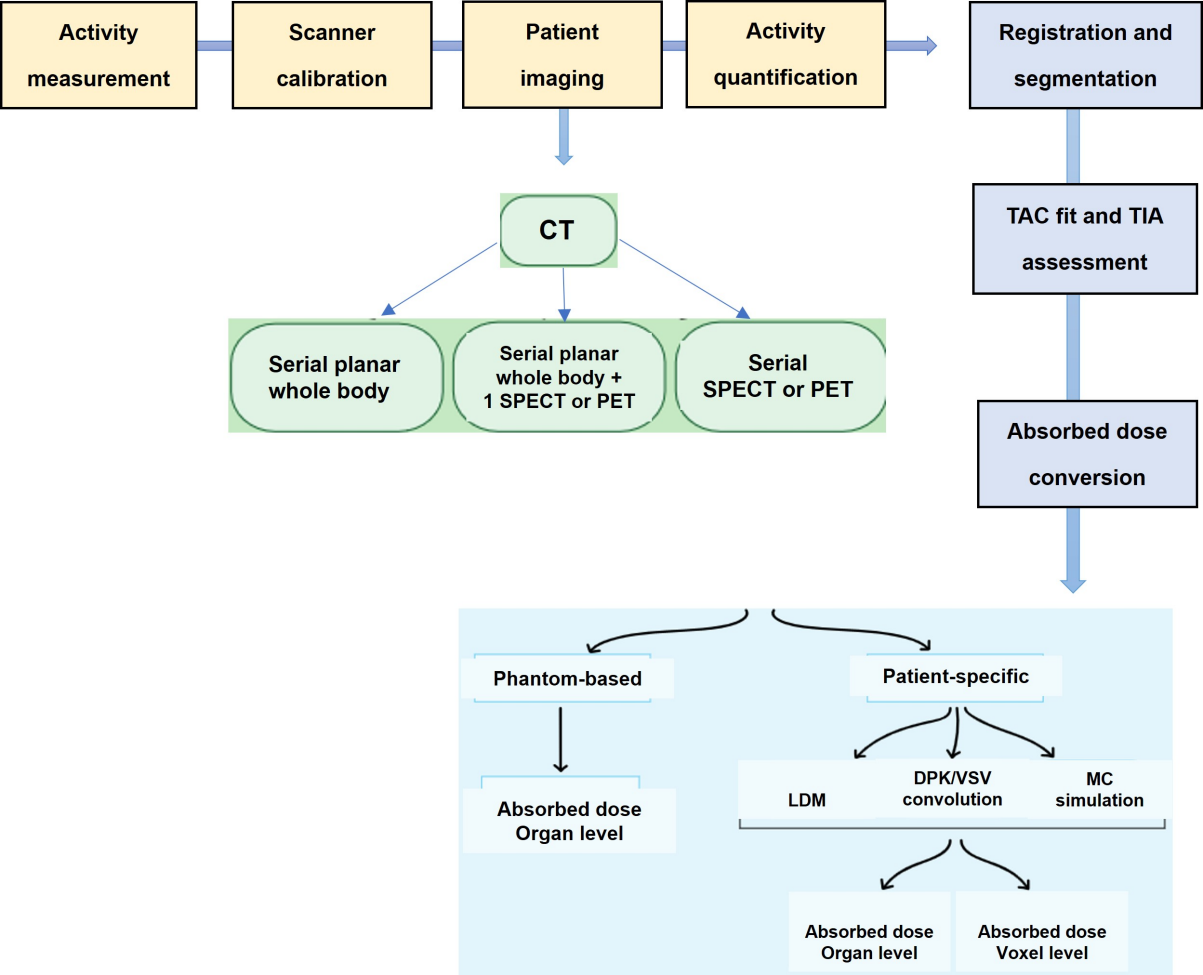
Schematic clinical dosimetric workflow.

Voxel-based dosimetry technique was developed that takes into account the non-uniform activity distribution. This includes dose point kernels and voxel S-value (VSV) approaches. The dose point kernel represents radial absorbed dose in a homogeneous aqueous medium when there is an isotropic point source at that location VSV is a voxel-level MIRD schema where sources and destinations are defined at the voxel level and then calculated in a 3D voxel matrix consisting of the aqueous medium. For more accurate individual dosimetry, voxel-based dosimetry is defined based on direct MC simulation. The MC simulation generates and tracks particles at the voxel level, calculates the stored energy, and absorbed dose at the voxel level (Figure 16).

The comparison between Ac-225 and Lu-177 dosimetry is essential due to the significant differences in their physical, chemical, and radiobiological properties, as well as the distinct challenges each presents. Ac-225, an alpha-emitter, offers high linear energy transfer (LET) radiation, which is highly effective for targeting micrometastases and single cancer cells. However, its complex decay chain, associated recoil effects, and redistribution of daughter isotopes pose substantial challenges for accurate dosimetry. On the other hand, Lu-177, a beta-emitter, provides more predictable energy deposition with established imaging capabilities using gamma emissions. These properties enable straightforward dosimetry using well-established macrodosimetric methods. Despite Lu-177's reliability in clinical applications, Ac-225 holds unique potential for treating specific cancer types where high LET radiation is beneficial. Hence, understanding the key differences and challenges in dosimetry between these radionuclides is crucial for optimizing therapeutic outcomes and advancing targeted radionuclide therapies.

##### a. Physical Properties

- Ac-225: An alpha-emitting radionuclide with high linear energy transfer (LET) radiation. Its decay chain produces multiple alpha particles and beta emissions, which contribute to its therapeutic efficacy but complicate dosimetry due to daughter redistribution and recoil effects 113.
- Lu-177: A beta-emitting radionuclide with low LET radiation, which allows for more predictable energy deposition over longer ranges. It emits gamma rays suitable for imaging, thus enabling more straightforward dosimetry 114.

##### b. Imaging and Quantification

- Ac-225: Alpha emissions are not directly imageable using standard nuclear imaging modalities. Indirect imaging relies on surrogate isotopes (e.g., Ac-227) or daughter radionuclides (e.g., Bi-213), which have different biodistribution profiles, leading to uncertainties in dosimetry 115.
- Lu-177: Gamma emissions at 113 keV and 208 keV are readily detected by SPECT, allowing accurate post-treatment imaging and quantification. Image-based dosimetry is well-established due to reliable gamma-ray detection 116.

##### c. Recoil Effects and Daughter Redistribution

- Ac-225: Alpha decay causes recoil of the parent nucleus, which can result in the release of daughter isotopes from the target tissue or carrier molecule, altering the dose distribution 115. Redistribution of daughters like Bi-213 and Pb-211 into off-target organs, such as the kidneys, complicates dose estimates and increases toxicity risks 113.
- Lu-177: No significant recoil effects are observed, and the radionuclide remains localized, resulting in predictable dose distribution. Established radiopharmaceuticals, such as Lu-177-DOTATATE, have well-characterized biokinetics and dosimetry 117.

##### d. Dosimetry Methods

- Ac-225: Requires complex modeling due to the contribution of multiple alpha particles and beta emissions from daughter nuclides. Microdosimetry approaches are often used to estimate the absorbed dose at the cellular level due to high LET and short alpha-particle ranges 118.
- Lu-177: Dosimetry is based on well-established macrodosimetric methods using quantitative SPECT imaging and standard MIRD (Medical Internal Radiation Dose) models 119.
- Personalized dosimetry using voxel-based approaches is increasingly used for optimizing therapeutic efficacy 120.

##### e. Clinical Implications

- Ac-225: Suitable for targeting micrometastases and single cancer cells due to the localized high LET radiation. Dosimetry challenges limit widespread clinical application despite promising therapeutic outcomes 121.
- Lu-177: Widely used in targeted radionuclide therapy for neuroendocrine tumors and prostate cancer. Established dosimetry protocols facilitate clinical translation and regulatory approval 114.

#### ^177^Lu/^225^Ac-DOTATATE dosimetry

Currently, there is no established standard dosimetry for ^177^Lu-DOTATATE in clinical practice. Typically, patients receive a fixed-activity regimen of 7.4 GBq in four therapeutic infusions, regardless of how the radionuclide is distributed in their body, as long as they can tolerate the full dose. Although it is possible to perform ^177^Lu-DOTATATE imaging during each therapy cycle, dosimetry can be utilized to estimate the optimal amount of activity to be injected in subsequent cycles. Despite observing a dose-response for ^177^Lu-DOTATATE through dosimetry analysis, the NETTER-1 prospective trial, which is considered a clinically relevant trial, did not utilize or report dosimetry data to guide treatment decisions. Some smaller trials that did use dosimetry to guide therapy did not find a significant correlation between tumor response outcomes and the administered activity 122. The upcoming COMPETE trial involving ^177^Lu-DOTATOC (ClinicalTrials.gov no. NCT03049189) is set to utilize dosimetry data from a significant number of participants, potentially providing valuable insights into the practical application of this method. Furthermore, the forthcoming DOBATOC trial (ClinicalTrials.gov no. NCT04917484) aims to evaluate the effectiveness of dosimetry in determining treatment dosage as opposed to using standard fixed-dose regimens. With the increasing experience in somatostatin receptor-targeted radionuclide therapy, more intricate situations like prolonged or repeated treatment cycles have become apparent. A particular study employed dosimetry to extend the cycles of ^177^Lu-DOTATATE until patients reached a specified renal dose threshold (23 Gy) or encountered other reasons for discontinuation, such as bone marrow toxicity or disease progression. Patients who tolerated higher doses demonstrated improved response rates and survival outcomes, though selection bias was a confounding factor 123. Dosimetry holds great potential in the future for adjusting dosage regimens and predicting the patients who would benefit from additional therapy cycles. This aspect, which is currently lacking in the existing literature, remains to be explored in future trials of targeted radionuclide therapy for NETs and other types of cancer. In order to predict dosimetry for ^225^Ac-DOTATATE, dosimetric data from ^177^Lu-DOTATATE was utilized. The time-activity curves for ^177^Lu-DOTATATE were adjusted to account for the physical half-life of ^177^Lu and were then employed for predictive dosimetry for ^225^Ac. To maintain equilibrium, residence times were estimated for all daughter isotopes in the ^225^Ac chain. Furthermore, specific S-values were taken into consideration for each daughter isotope to accurately evaluate their contributions to the overall dose.

#### ^177^Lu/^225^Ac-PSMA-617 dosimetry

In the same manner as the management of ^177^Lu-DOTATATE, a fixed activity regimen is utilized for the administration of ^177^Lu-PSMA-617 in current clinical practice. The implementation of dosimetry could provide significant prognostic information for radionuclide therapy in prostate cancer. Although dosimetry has been conducted for ^177^Lu-PSMA-617 during each cycle in experimental settings, demonstrating the ability to predict PSA response, it has not been employed to direct dose administration for prostate cancer RLT and is not documented in prominent prospective trials such as TheraP or VISION 124, 125. Ongoing endeavors are being made to establish uniform dosimetry techniques for radiopharmaceutical therapies. In this regard, collaborative guidelines have been developed by the EANM and the MIRD society, specifically focusing on the dosimetry of ^177^Lu using quantitative SPECT. These guidelines have been successfully implemented in clinical trial settings 124. Dosimetry is commonly required for the implementation of EBRT in clinical settings. Some nations are now recognizing the importance of dosimetry in conjunction with radiopharmaceuticals. Nevertheless, there is an ongoing discussion about whether dosimetry calculations should be mandatory in the clinical administration of ^177^Lu-DOTATATE and ^177^Lu-PSMA-617, as well as the advantages of tailoring therapeutic doses based on individual patient needs using dosimetry. A significant challenge in dosimetry for RLT is the limited ability to effectively image α-particle emitters. However, it is still possible to estimate dosimetry by utilizing a diagnostic surrogate radionuclide that emits γ rays, although it should be noted that changing the radionuclide could impact biodistribution 126. These measurements have the potential to customize treatment by optimizing the correlation between dosage and response, all the while ensuring the safety of vital organs. It is of utmost importance to continue striving towards evaluating the influence of dosimetry on patient outcomes and establishing uniform techniques that can facilitate dosimetry in different medical facilities. These endeavors are essential to fully realize the potential advantages of dosimetry. The dosimetric data of ^177^Lu-PSMA ligands were transformed into predictive dosimetry for ^225^Ac-PSMA ligands, under the assumption of a comparable uptake pattern governed by the PSMA carrier, following the methodology previously described by Kratochwil *et al.* 121, 127. The TACs for ^177^Lu-PSMA were modified to accommodate the physical half-life of ^177^Lu, and the resulting biological TACs were employed for the predictive dosimetry of ^225^Ac. To ensure equilibrium within the decay chain and assume no translocation during decay between subsequent disintegrations, the same residence time estimated for ^225^Ac was applied to all the daughter isotopes in the ^225^Ac chain. Additionally, specific S-values were taken into account for each daughter isotope to accurately evaluate their respective dose contributions. Drawing from existing literature, an RBE factor of 5 was utilized to assess the α-particle dose contribution in comparison to the γ and β emissions 128.

#### Whole-body voxel-based internal dosimetry using deep learning

Personalized medicine presents a new approach that seeks to enhance the effectiveness of healthcare and lower expenses. It holds great potential for tailoring diagnosis and treatment to each individual, thereby offering improved outcomes 129-132. Precision medicine aims to shift from the prevailing one-size-fits-all approach to a personalized model. Within the realm of nuclear medicine, the calculation of dosage assumes a crucial role in aligning with this paradigm 107. The precise determination of individualized dosages is crucial within this framework as it allows for the optimization of clinical protocols while reducing the risk of radiation-related side effects 133. At present, the monitoring of patient dose in clinical settings frequently depends on simplified models, such as those obtained from the MIRD formalism 134. The traditional MIRD method calculates organ-level dosimetry using time-integrated activity and radionuclide S-values to estimate the average absorbed dose per radioactive decay. This approach assumes consistent activity distribution within organs and ignores individual anatomical differences. To address variability in anatomy, newer techniques incorporate patient-specific computational models 135-138.

Furthermore, there have been advancements in voxel-based dosimetry methods, including the dose point kernel and VSV techniques 134. Unlike probabilistic approaches, the dose point kernel method 139 computes the radial absorbed dose profile surrounding an isotropic point source within a uniform water medium 140, 141. The MIRD schema at the voxel level is described as a 3D voxel matrix that shows the average absorbed dose to a target voxel for each unit of activity in a source voxel within an infinite homogeneous medium using MC simulations. Nevertheless, when performing voxel-based dose calculations, it is essential to take into account the non-uniform distribution of activity of the radiotracer, as well as the heterogeneity of the medium, which includes various material compositions such as lung, soft tissue, and bone, that are often overlooked. To overcome this challenge, direct MC simulations, recognized as the standard for establishing a dependable dose calculation framework in a clinical environment, enable the precise calculation of a complete body dose map 142, 143.

**Figure 17:**
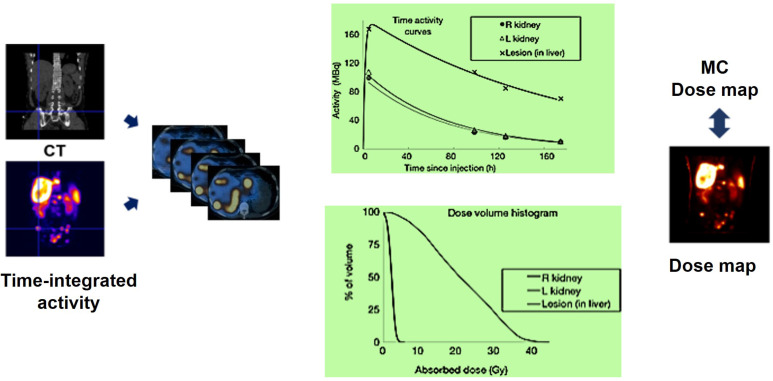
Sample dose volume histograms (DVHs) and lesion absorbed dose map corresponding to a patient imaged at 4 time points after cycle 1 of standard (7.4 GBq) ^177^Lu-DOTATATE RLT. SPECT/CT images at each time point were input to a Monte Carlo dosimetry code and the corresponding dose-rate maps were integrated to derive the absorbed dose map 147.

MC simulation considers non-uniform activity distribution and patient-specific anatomical features, but it is limited by computational workload. Past research has explored MC simulations for personalized dosimetry in nuclear medicine 144-146. The MC simulator uses hybrid PET/CT or SPECT/CT images to simulate radiation energy deposition from injected radiotracers, considering patient anatomy and voxelwise activity distribution (Figure 17).

Studies aim to balance voxel-scale dosimetry accuracy with computational efficiency 148, 149. Khazaee Moghadam and colleagues proposed a method utilizing tissue-specific dose point kernels on a stylized phantom 150. Building on this concept, Lee *et al.* extended the methodology by applying it to real patient data 151. They incorporated various material densities into internal dose calculations, creating a range of voxelwise S-value kernels for different human body tissues. This method allowed for the generation of multiple voxel-scale dose maps similar to MIRD calculations. Each density-specific dose map was multiplied by the corresponding binary mask from CT-based segmentation to compute the final dose map. While this approach enhances dosimetry accuracy compared to single voxel S-value methods, it assumes energy depositions primarily occur due to self-absorption, introducing potential errors at tissue boundaries. The importance of precise patient-specific dosimetry is highlighted by advancements in targeted radionuclide therapy and theranostic imaging 107. MC simulation is considered the most accurate method for personalized dosimetry and is the gold standard for research, but its use in clinical settings is limited by its heavy computational requirements. Deep learning has emerged as a promising tool in computer vision and image processing, outperforming traditional methods in medical image analysis for PET and SPECT imaging. It excels in tasks such as attenuation and scatter correction 152-157, low-count image reconstruction 158-162, and automated image segmentation 138, 163-165.

Recently, deep learning techniques have been increasingly used in radiation dose estimation. Mardani *et al.* have introduced a new method using a multi-layer convolutional auto-encoder to predict dose distribution in external beam radiation therapy 166. Nguyen *et al.* utilized a U-Net structure in their study on clinical treatment plan optimization, to improve the quality and consistency of treatment plans while also decreasing the computational time required 167. Ma *et al.* successfully employed a deep learning approach to extract isodose characteristics in the context of modulated arc therapy treatment plans 168. Kearney and colleagues introduced a three-dimensional fully convolutional algorithm designed specifically for predicting doses in prostate stereotactic body radiotherapy patients 169. Effective training of a deep learning algorithm relies on a well-defined ground truth as an essential ingredient 170.

The earlier influential studies used MC dosimetry as a substitute to establish ground truth for training the networks. However, this approach may result in inaccuracies due to simplifications in physical models 171. Lee *et al.* employed a U-Net deep neural network architecture to address this constraint, utilizing training data derived from MC simulation for internal dosimetry purposes 172. Gotz *et al.* used a modified U-Net network to estimate dose maps for patients given ^177^Lu-PSMA ligands. They input CT images and static PET images into the network to predict a 3D dose map 173. The training datasets in this research included a two-channel input: CT images for patient-specific density maps and MIRD-based voxel-scale dose maps from SPECT images. Ground truth was obtained from direct MC simulations. Previous studies used deep learning networks trained with whole-body dose maps from direct MC simulations, but creating a comprehensive training dataset was challenging due to the computational intensity of MC calculations. As a result, these studies either used a limited number of training samples or made approximations that could impact model accuracy.

## Procedure guidelines or recommended practice

The establishment must have enough seating and essential amenities to ensure patient well-being. Chair use is typically limited to three infusions per day. We have expertise in chair breakdowns, radiation monitoring, and preparation procedures. Safety measures are customized to contain radioactivity and depend on the therapy and radionuclides used. For example, administering α-particle emitters like ^223^Ra dichloride is straightforward because α-particles travel a very short distance (<100 μm) in most substances (Table 6) 174-176.

Therefore, minimal shielding and personal protective equipment are required for α-emitting RLT, allowing it to be administered in a clinic or office setting if desired 177, 178. Conversely, β-emitting RLT, like ^177^Lu vipivotide tetraxetan (Pluvicto) for prostate cancer, emits γ-photons in addition to β-particles, requiring more complex considerations for the facility 179, 180, especially in terms of potential daily exposure risks for medical personnel. A dedicated hot lab for storing and preparing Pluvicto doses is necessary, and specific therapy rooms must be designated. While some nuclear medicine centers have lead-shielded private compartments for infusion chairs to enhance radiation protection, shielded patient rooms are not mandatory for Pluvicto administrations. However, patients receiving Pluvicto treatment should be isolated from others.

### Patient scheduling

Patients scheduled for RLT need to be informed about potential treatment delays due to myelosuppression. Timely completion of necessary blood work is crucial to avoid cancellations and wasted doses. The complexity of maintaining timely administration is compounded by the decay profile of radioactive material and fixed treatment intervals. Supply chain interruptions for radiopharmaceuticals can also cause delays. Patients should be informed about possible schedule adjustments due to adverse events or supply chain disruptions. Providing logistical support can improve patient scheduling and access to RLT. The medical management plan for oncology therapies involves imaging, treatment, blood work, and follow-up appointments overseen by the prescribing clinician. Advanced Practice Providers can help manage RLT schedules and monitor patient progress. Collaboration between the oncology team and transfusion facility is essential for patients dependent on transfusions. Computerized workflow applications can optimize scheduling and prevent inefficiencies. Accurate forecasting is key to ensuring appropriate treatment 37, 179.

Imaging and RLT centers depend on a consistent supply of radiopharmaceutical agents, which are mainly produced in limited facilities like cyclotrons and nuclear reactors. Effective communication with suppliers is crucial due to the ordering timelines, with ^177^Lu-based RLTs requiring orders to be placed at least two weeks in advance 37, 179. Managing these timelines adds complexity to the supply chain, requiring additional personnel to promptly process orders through the Novartis Advanced Accelerator Applications Radiopharmaceutical Order Management Environment. This system helps minimize wastage, ensure timely patient care, and reduce the need for rescheduling. For Pluvicto, RLT centers coordinate their orders and supply based on a dosage regimen of 7.4 GBq administered every 6 weeks for up to 6 cycles via IV infusion 181.

### Patient admission

**Figure 18:**
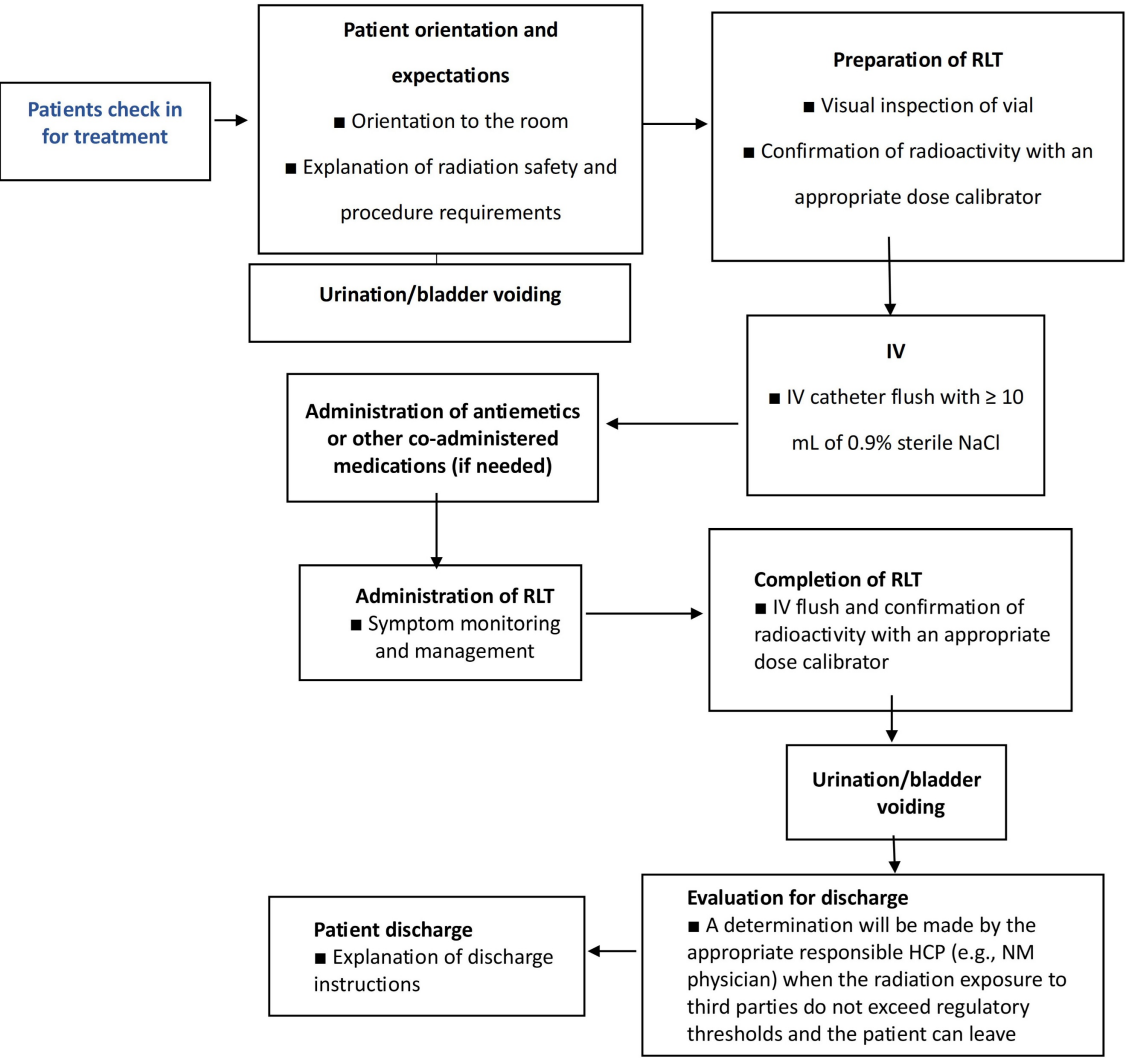
Patient's journey on treatment day. a. HCP, healthcare provider; IV, intravenous; NaCl, sodium chloride; NM, nuclear medicine; RLT, radioligand therapy. Advise patients to remain well hydrated and to urinate.

Nurses play a crucial role in ensuring the smooth implementation of RLT treatments. Prior to the treatment day, nurses must confirm the schedule with the patient and engage in a comprehensive discussion regarding the planned procedures. This pre-treatment communication aims to educate and prepare the patient, addressing any incontinence requirements (such as self-catheterization or the use of incontinence pads) to minimize potential radiation safety risks. Additionally, this conversation can also involve coordinating travel arrangements to the center, with certain centers assisting in accommodations the night before and/or after the treatment. The establishment of an efficient workflow is vital for the safety of both staff and patients, ultimately enhancing the patient's overall treatment experience (Figure 18). To ensure effective education, treatment, and supervision in RLT, dedicated staff should assist patients in getting acquainted with the therapy room, explaining safety protocols, and answering any questions.

### Radioligand preparation

Prior to administration, a nuclear medicine technologist thoroughly examines the radioligand preparation to ensure the absence of any particulate matter. Subsequently, the technologist utilizes a dose calibrator to accurately measure the activity, guaranteeing that the patient receives the prescribed dose with precision. Following this, the radioligand is prepared for administration. If the radioligand is contained in a vial, it must be prepared in a hot lab before it can be transferred to a shielded syringe if the syringe method of administration is chosen 181. In the case of Pluvicto, the standard approved activity of 7.4 GBq is typically administered to most patients 181. However, this dosage can be adjusted if there are any risk factors that may pose a potential toxicity concern 127, 182.

### Administration

Prior to administration, it is crucial to perform suitable imaging examinations, comprehensive blood counts, and assessments of kidney function. The technique of RLT administration differs based on the particular RLT being employed. The equipment necessary for RLT administration, in adherence to radiation safety protocols, encompasses tongs, shielded barriers equipped with leaded glass, syringe shields, kits for handling radioactive material spills, and Geiger counters 179. It is essential to have a treatment room with a patient restroom available at all times during the treatment period to accommodate frequent voiding needs. Patients who may experience incontinence should follow institutional guidelines treating excreta as radioactive waste. Moreover, all pre-medications and concomitant medications must be easily accessible. In the instance of Pluvicto, an antiemetic like oral ondansetron can be given 30-60 minutes before infusion and on days 2 and 3 of each treatment cycle 182. Per the recommendations outlined by the EANM, corticosteroids may be administered starting one day prior to treatment and continued for multiple days post-treatment in individuals with metastases who are at risk of experiencing painful or obstructive swelling following RLT 182, 183. In contrast to the use of amino acids for renal protection during Pluvicto treatment, which is necessary for neuroendocrine tumors treated with ^177^Lu-DOTATATE, establishing proper venous access is crucial when administering RLT 181, 184. The typical method of administering Pluvicto involves slowly pushing it through an IV catheter prefilled with 0.9% sterile sodium chloride solution over a period of approximately 1-10 minutes. Other methods of administration, such as the gravity method or the vial method, can also be used. To minimize the risk of extravasation, a saline flush of at least 10 mL is required before and after the infusion 184.

Patients receiving Pluvicto treatment require close monitoring by a nurse under the supervision of the attending physician to address any issues efficiently. It means that immediate issues may arise during or after Pluvicto treatment, necessitating close monitoring. Pluvicto, being a radioligand therapy, can potentially cause acute side effects that require prompt management. These issues may include:

a. Infusion-Related Reactions: Patients might experience nausea, vomiting, or hypersensitivity reactions during or shortly after infusion.

b. Gastrointestinal Symptoms: Diarrhea or abdominal discomfort could occur, which may need immediate symptomatic treatment.

c. Radiation Safety Concerns: Handling potential radioactive contamination or addressing immediate radiation-related issues may also require attention.

Close monitoring ensures that any acute complications are promptly identified and managed, minimizing risks and improving patient safety during treatment.

Potential consequences may arise 3-4 weeks after therapy 184. The most common side effects that are not immediate issues of Pluvicto are listed below 185:

Hematological Toxicity: Pluvicto can lead to myelosuppression, manifesting as low blood counts (e.g., anemia, neutropenia, thrombocytopenia), which might require urgent intervention in severe cases. The initial two cycles of PSMA-RLT were generally well tolerated in terms of hematological effects. The degree of both qualitative and quantitative impairment of the bone marrow seems to correlate significantly with the burden of osseous tumors. Notably, only these patients with extensive bone involvement and a lack of therapeutic response experienced severe hematological adverse events, alongside a marked reduction in hematological parameters. This suggests that in individuals with mCRPC, a lack of response to PSMA-RLT may be a significant factor contributing to bone marrow impairment during the early stages of treatment. Renal Function Impairment: As the kidneys are primarily responsible for excreting the radioactive component, monitoring for signs of acute kidney injury is crucial.

Common adverse reactions with an incidence of at least 20% include fatigue, dry mouth, nausea, anemia, decreased appetite, and constipation. Common laboratory abnormalities with an incidence of at least 30% include reductions in lymphocytes, hemoglobin, leukocytes, platelets, calcium, and sodium 181. Adverse events, such as vomiting and diarrhea are frequently reported as well. Patients with extensive bone metastases may encounter bone marrow toxicity; however, it is important to note that while a high osseous tumor burden is a risk factor, bone marrow toxicity can also occur in patients without bone metastases 184. Cytopenia of clinical significance following Pluvicto treatment can be managed by administering transfusions, and the subsequent treatment cycle can be delayed until blood counts improve. Patients with bone metastasis that causes symptoms may experience a temporary increase in pain lasting for 3-7 days, which can be effectively managed with pain medication, steroids, and/or non-steroidal anti-inflammatory drugs. To monitor these symptoms and toxicities in prostate cancer patients undergoing Pluvicto treatment, nurses and physicians can utilize patient-reported outcome measures specifically designed for this type of therapy, such as the recently developed Functional Assessment of Cancer Therapy-Radionuclide Therapy (FACT-RNT) 186.

### Patient discharge

Following the completion of treatment, the radiation safety officer (RSO) is responsible for assessing radiation levels at a distance of 1 meter from the patient. If the anticipated dose to any other individual is projected to be under 30 μSv/h, patients are permitted to travel long distances or proceed to the airport for their journey home, in accordance with our standard operating procedure. Discharge criteria for patients treated with ^177^Lu-based therapies vary significantly across countries due to differences in regulatory frameworks and clinical practices. For example, in the United States, it is standard practice to discharge patients after ^177^Lu-based therapy without requiring the Radiation Safety Officer (RSO) to measure radiation levels at a distance of 1 meter from the patient. Conversely, other countries may mandate stricter discharge protocols, including radiation monitoring, to comply with local radiation safety regulations. In an analysis by Demir *et al.*, the mean dose rate at 1 m, 4 hours, and 6 hours post-treatment for 23 patients receiving Pluvicto was 23 ± 6 μSv/h and 15 ± 4 μSv/h, respectively 187. Although ^177^Lu emits relatively low levels of γ-radiation, the radiation protection guidelines for this isotope are not as strict as those for isotopes with higher γ-emission, such as iodine-131 188. Patients, caregivers, and healthcare professionals should receive detailed contamination control instructions during and after clinic visits. Patients must be aware of potential radioactive contamination from bodily fluids, as about half of Pluvicto activity is excreted in urine within four hours of treatment 189. Patients should practice good bathroom hygiene and stay hydrated at home to achieve optimal clearance, given the typical duration of discharge 190. Close contact with adults should be limited during the 48 hours following therapy. Contact with children and pregnant women should be avoided for at least 168 hours. These general guidelines aim to minimize radiation exposure while accounting for international variability in practices and ensuring compliance with radiation safety standards.

It is recommended to maintain separate sleeping arrangements from children for a week and from pregnant women for two weeks. Additionally, refraining from engaging in sexual activity is recommended for a duration of one week. Patients should promptly report any significant adverse events, especially myelosuppression symptoms, to the clinic and inform non-oncology healthcare providers about their Personalized Radiation Therapy. Coordinated care inquiries should be directed to the oncology care team, as regular follow-up calls by the designated healthcare provider are crucial, given that some patients may not recognize severe adverse events.

### Perspectives

As theranostics emerges as a pivotal approach in nuclear medicine and precision medicine, the establishment of standardized training programs becomes increasingly imperative 21. These programs not only ensure safe and effective practice but also pave the way for future advancements. By fostering multidisciplinary collaboration among nuclear medicine professionals, qualified specialists, and healthcare experts, new technologies and treatment modalities can be seamlessly integrated. Adhering to guiding principles will be essential for maintaining high standards, optimizing outcomes, and driving innovation 191. Furthermore, specialized centers for radiotheranostic services are crucial, particularly in regions with limited access to innovative treatments. These centers play a vital role in bridging disparities between developed and developing nations, addressing challenges such as financial constraints and resource limitations 192. Investing in professional training in dosimetry, radiochemistry, and radiopharmacy is key to enhancing treatment access, ensuring quality, and building specialized expertise and facilities in theranostics. The potential of combination theranostics with other targeted treatments may maximize synergistic benefits of personalized medicine but this requires careful examining through clinical trials 193.

## Conclusion

The survival and quality of life for numerous patients are being significantly improved by the expanding landscape of radiotheranostics. This advancement has sparked the investigation and creation of new agents, as well as the broadening of indications for approved therapies. Although RPT represents a significant step towards precision medicine, there is still potential for further progress by gaining a deeper understanding of patient-specific factors that contribute to organ toxicity and tumor-related elements that influence treatment response. The customization of treatment for individual patients offers the potential to move closer to a paradigm of personalized disease management. In the current investigation, we present a thorough examination of the involvement of various radioisotopes in the induction of direct and indirect DNA damage, as well as their influence on the initiation of DNA repair mechanisms in cancer cells. Current data indicates that high-energy α-emitter radioisotopes have the potential to directly impact the DNA structure by causing ionization, resulting in the creation of ionized atoms or molecules. This ionization process predominantly leads to the formation of irreparable and intricate DSBs. On the other hand, the majority of DNA damage caused by β-emitting radioisotopes is indirect, as it involves the production of free radicals like ROS and subsequent chemical reactions. Beta particles themselves can also physically interact with the DNA molecule, resulting in SSBs and potentially reversible DSBs. Drawing on real-world experience, we offer practical guidance for the effective integration of RLT into current clinical practices. As the field of RLT continues to evolve rapidly, robust RLT programs will need to adapt and refine the provided guidance in the future.

While this review provides a broad overview of multiple aspects of radiotheranostics, each section is supported by targeted references for readers who wish to explore specific topics in greater depth. The aim was that this structured approach facilitates both a comprehensive understanding and an easy transition to more detailed investigations in the field.

## Figures and Tables

**Figure 10:**
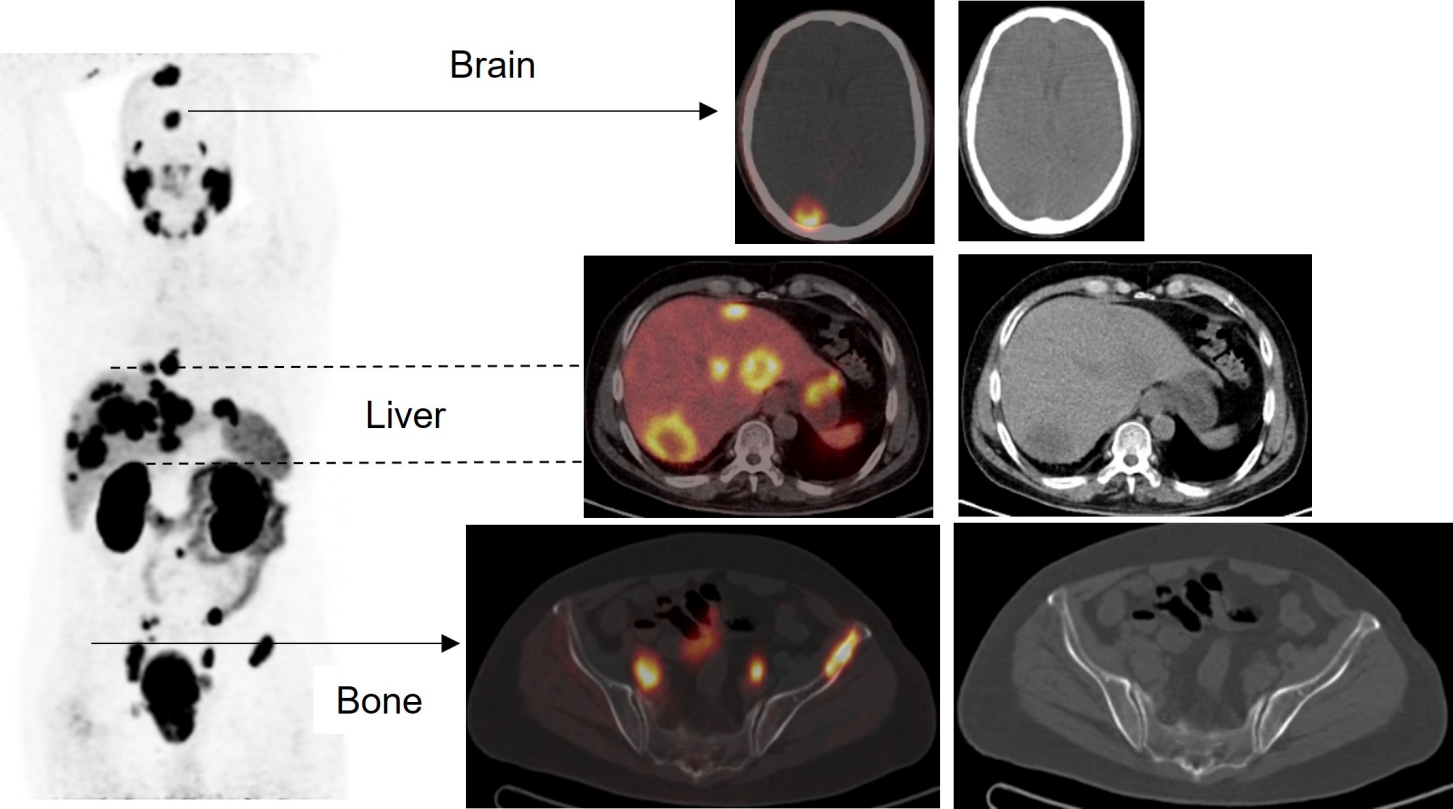
A new case of prostate cancer (poorly differentiated carcinoma) with a PSA level of 21 has been referred for staging. The staging evaluation revealed the presence of multiple lymph nodes with PSMA uptake in the para-aortic, common iliac, external, and internal iliac chains, measuring up to 28mm with a SUV_max_ of 15.10. Additionally, the liver exhibited multiple PSMA-avid masses in both lobes, measuring up to 52mm with an SUV_max_ of 16.16. The prostate gland appeared enlarged and lobulated, displaying significant inhomogeneous PSMA uptake. There is a likelihood of invasion into the infero-posterior wall of the urinary bladder, with an SUV_max_ of 18.07. Metastases were observed in various locations, including the skeletal system, both liver lobes, bilateral lung field, and brain. Notably, there was intense uptake in the salivary glands.

**Table 1:** Characteristics and applications of emitted radiation (α, β-minus and γ radiation).

Type of Emission	LET	Range	Effect	Application
α radiation	+++	+	Dense, highly localized damage	Therapy
β-minus radiation	++	++	Intermediate range and damage	Therapy
γ radiation	+	+++	Sparsely ionizing and long range	Imaging

**Table 2:** PSMA expression score and eligibility for RPT according to the PROMISE V2 criteria.

Score	Uptake Relative to Internal Reference	Eligibility for RPT
0	≤Blood pool	No
1	≤Liver and > blood pool	No
2	≤Parotid gland and > liver	Yes
3	>Parotid gland	Yes

Organs that exhibit elevated physiologic uptake are more susceptible to the harmful effects of radiation. An example of this is the occurrence of dry mouth, known as xerostomia, which was reported as a prevalent side effect in 38.8% of patients undergoing ^177^Lu-PSMA ligands treatments in the VISION trial (Figure 10) 32, 36.

**Table 3:** Example of emerging molecular targets in clinical trials (NET).

Molecular Targeting Mechanism	Trial Registration No. and Agents	Type of Tumor
	NCT02609737: ^68^Ga-DOTA-JR11/^177^LuDOTA-JR11	NET
	NCT02592707: ^68^Ga-OPS202/^177^Lu-OPS201	NET
Somatostatin receptor antagonist	NCT04997317: ^177^Lu-satoreotide	Meningiomas
	NCT05017662: ^177^Lu-IPN01072	NET, long-term surveillance for secondary malignancies
	NCT05359146: ^161^Tb-DOTA-LM3	NET

**Table 4:** Software packages for phantom-based dosimetry.

Name	Availability	Decay Data	Number of Radionuclides	Phantoms	Specific Organ Models
OLINDA/EXM 1 (2004) Olinda/EXM® 2.0	Distributed by Vanderbilt University, presently Withdrawn from the market	RADAR website	Over 800	Cristy and Eckerman + pregnant female series	Peritoneal cavity, prostate gland, head and brain, kidney and spheres
Organ DosimetryTM with	Distributed by Hermes Medical	RADAR website	Over 1000	RADAR phantoms + animal phantoms	Peritoneal cavity, prostate gland, head and brain, kidney and spheres
IDAC 2.1 (2017)	Free	ICRP 107	1252	ICRP 110	Spheres
3D-RD-S (2020)	Distributed by Rapid, LLC	ICRP 107	1252	ICRP 110 and ICRP 143	Spheres
MIRDcalc (2021)	Free	ICRP 107	333	ICRP 110, ICRP 143 and weight-based phantoms	Spheres

**Table 5:** Main commercial software packages for patient-specific dosimetry.

Name	Manufacturer	Dose Conversion Method	Supported Therapy Radionuclides	CE/FDA Approval
SurePlan™ MRT	MIM Software Inc.	VSV	^177^Lu, ^131^I	CE/FDA
Planet® Dose	DOSIsoft	VSV/LD M	^177^Lu, ^131^I	CE/FDA
Voxel DosimetryTM	Hermes Medical solutions	Semi-MC	^68^Ga, ^123^I, ^131^I, ^111^In, ^177^Lu, ^99m^Tc, ^90^Y, ^89^Zr, ^223^Ra, ^166^Ho	CE/FDA
QDOSE®	ABX-CRO	VSV	^11^C, ^15^O, ^18^F, ^44^Sc, ^64^Cu, ^68^Ga, ^86^Y, ^89^Zr, ^90^Y, ^124^I, ^89^Sr, ^99m^Tc, ^111^In, ^131^I, ^153^Sm, ^166^Ho, ^177^Lu, ^186^Re, ^188^Re	CE
SurePlan™ LiverY90	MIM Software Inc.	VSV/LD M	^90^Y microspheres	CE/FDA
Planet®	DOSIsoft	Dose VSV/LD M	^90^Y microspheres	CE/FDA
Hybrid3DTM SIRT	Hermes Medical solutions	LDM	^90^Y microspheres	CE/FDA
Simplicit90YT M v2.4	Mirada Medical	LDM	^90^Y microspheres	CE/FDA
VelocityTM Varian RapidSphere v4.1	Varian	DPK/LD M	^90^Y microspheres	CE/FDA
Q-Suite v2.0	QUIREM Medical BV	LDM	^166^Ho microspheres	CE

**Table 6:** Radiation safety requirements for radionuclides used in prostate cancer treatment.

Element	Tissue penetration (mm)	Radionuclide half-life	Specific material handling needs required to minimize radiation exposure
*α-emitting*			Standard personal protection equipment, including mask, gloves, laboratory coat, safety glasses/goggles
^223^Radium	< 0.1	11.4 d	
^225^Actiniuma	0.05-0.08	10.0 d	
^227^Thoriuma	0.05-0.08	18.7 d	
^211^Astatinea	0.05	7.2 h	
^212^Leada	0.06-0.09	10.6 h	
*β-emitting*			Shielding with an appropriate thickness of low atomic number (Z < 14) materials (e.g., Plexiglas) or aluminum Standard personal protection equipment, including gloves, laboratory coat, safety glasses/goggles
^131^Iodine	0.8	8.0 d	
^177^Lutetium	0.67	6.6 d	
^153^Samariumb	0.40	46.5 h	
^90^Yttrium	5.30	64.1 h	
^67^Coppera	0.6	2.6 d	
^161^Terbiuma	0.29	6.9 d	
